# Fast skeletal myosin binding protein-C expression exacerbates dysfunction in heart failure

**DOI:** 10.1007/s00395-026-01202-8

**Published:** 2026-08-05

**Authors:** James W. McNamara, Taejeong Song, Perwez Alam, Akhil Baby, Aleksandra Binek, Kalyani Ananthamohan, Rohit R. Singh, Michelle L. Nieman, Marloes van den Berg, Brent M. Cernyar, Sheryl E. Koch, Malina J. Ivey, Thomas L. Lynch, James T. Pearson, Jack Rubinstein, J.-P. Jin, John N. Lorenz, Jennifer E. Van Eyk, Onur Kanisicak, Sakthivel Sadayappan

**Affiliations:** 1 Division of Cardiovascular Health and Disease, Department of Internal Medicine, Center for Cardiovascular Research, University of Cincinnati, Cincinnati, OH USA; 2 Murdoch Children’s Research Institute, Royal Children’s Hospital, Melbourne, VIC 3052 Australia; 3 Victor Chang Cardiac Research Institute, Darlinghurst, NSW 2010 Australia; 4 Department of Cellular and Molecular Medicine, Sarver Heart Center, University of Arizona College of Medicine, Tucson, AZ USA; 5 Department of Pathology and Laboratory Medicine, University of Cincinnati College of Medicine, Cincinnati, OH USA; 6 Department of Genetic Engineering, School of Biotechnology, Madurai Kamaraj University, Madurai, India; 7 Cedars-Sinai Medical Center, Advanced Clinical Biosystems Research Institute, Smidt Heart Institute, Los Angeles, CA 90048 USA; 8 Department of Pharmacology and Systems Physiology, University of Cincinnati College of Medicine, Cincinnati, OH USA; 9 Department of Cell and Molecular Physiology, Loyola University Chicago, Maywood, IL USA; 10 Department of Cardiac Physiology, National Cerebral and Cardiovascular Center Research Institute, Suita-shi, Osaka, Japan; 11 Department of Physiology, Wayne State University School of Medicine, Detroit, MI USA; 12 Department of Biomedical Sciences, Cedars-Sinai Medical Center, Los Angeles, CA 90048 USA; 13 Novo Nordisk Foundation Centre for Stem Cell Medicine (reNEW), Murdoch Children’s Research Institute, Melbourne, VIC Australia; 14 Present Address: Discovery Protein Science, Amgen, South San Francisco, CA 94080 USA; 15 Present Address: Department of Biomedical Sciences, University of Missouri, Columbia, MO 65211 USA; 16 Present Address: Department of Physiology and Biophysics, University of Illinois at Chicago, 835 S Wolcott Ave., Chicago, IL 60612 USA; 17 Division of Basic and Translational Science, Department of Emergency Medicine, The Ohio State University Wexner Medical Center, Columbus, OH USA

**Keywords:** *Mybpc2*, *Mybpc3*, Myofilament, Heart failure, Transcomplementation

## Abstract

During heart failure (HF), gene and protein expression profiles undergo extensive compensatory and pathological remodeling. We previously observed that fast skeletal myosin binding protein-C (fMyBP-C) is upregulated in diseased mouse hearts. While fMyBP-C shares significant homology with its cardiac paralog, cardiac myosin binding protein-C (cMyBP-C), there are key differences that may affect cardiac function. However, the consequence of cardiac expression of fMyBP-C is unknown. Here, we aim to elucidate the impact of fMyBP-C expression on cardiac function and pathology. To determine the sufficiency of fMyBP-C to cause cardiac dysfunction, we generated cardiac-specific fMyBP-C over-expression mice. These mice were crossed into cMyBP-C null mice to assess the effect of fMyBP-C in the complete absence of cMyBP-C. Finally, fMyBP-C null mice underwent transverse aortic constriction (TAC) to define the requirement of fMyBP-C in HF. We confirmed the upregulation of fMyBP-C in several models of cardiac disease, including lineage tracing models. Low levels of fMyBP-C caused mild cardiac remodeling and sarcomere dysfunction. Exclusive expression of fMyBP-C in a HF model exacerbated cardiac pathology. Furthermore, reduction of fMyBP-C expression by shRNA in HF improved cardiac function. Following pressure overload, fMyBP-C null mice demonstrated greater resistance to cardiac decompensation. Mechanistically, our data suggest the differential regulation of the myosin super-relaxed state by cMyBP-C and fMyBP-C plays a contributing role. These findings suggest the elevated expression of fMyBP-C in diseased hearts is a pathological response. Targeted therapies to prevent upregulation of fMyBP-C may prove beneficial in the treatment of HF.

**Supplementary Information:**

The online version contains supplementary material available at https://doi.org/10.1007/s00395-026-01202-8.

## Introduction

Heart failure (HF) is a devastating global disease with growing prevalence that places extreme burden on patients, families, and communities [39]. While recent progress has improved outcomes, treatments remain insufficient for a disease of this scale [5]. There is thus an urgent requirement for the development of new and more effective therapies based on the underlying molecular mechanisms of cardiac disease.

During the onset of HF, there are dramatic alterations in transcriptional and proteomic networks in the heart [16, 26, 31, 35]. These alterations include activation of fetal [26, 33], pro-fibrotic [7, 44], immune [1, 22, 41], and pro-hypertrophic [10, 43] programs. While these networks are complex and variable, they ultimately converge on a common endpoint which results in pathologically diminished contractile function [9]. Accordingly, recent years have seen an increased focus towards the development of sarcomere-targeted therapies for the treatment of cardiac diseases [27]. Clearly, an in-depth understanding of the molecular alterations at the sarcomere is key to the success of such drug development. 

**Fig. 1:**
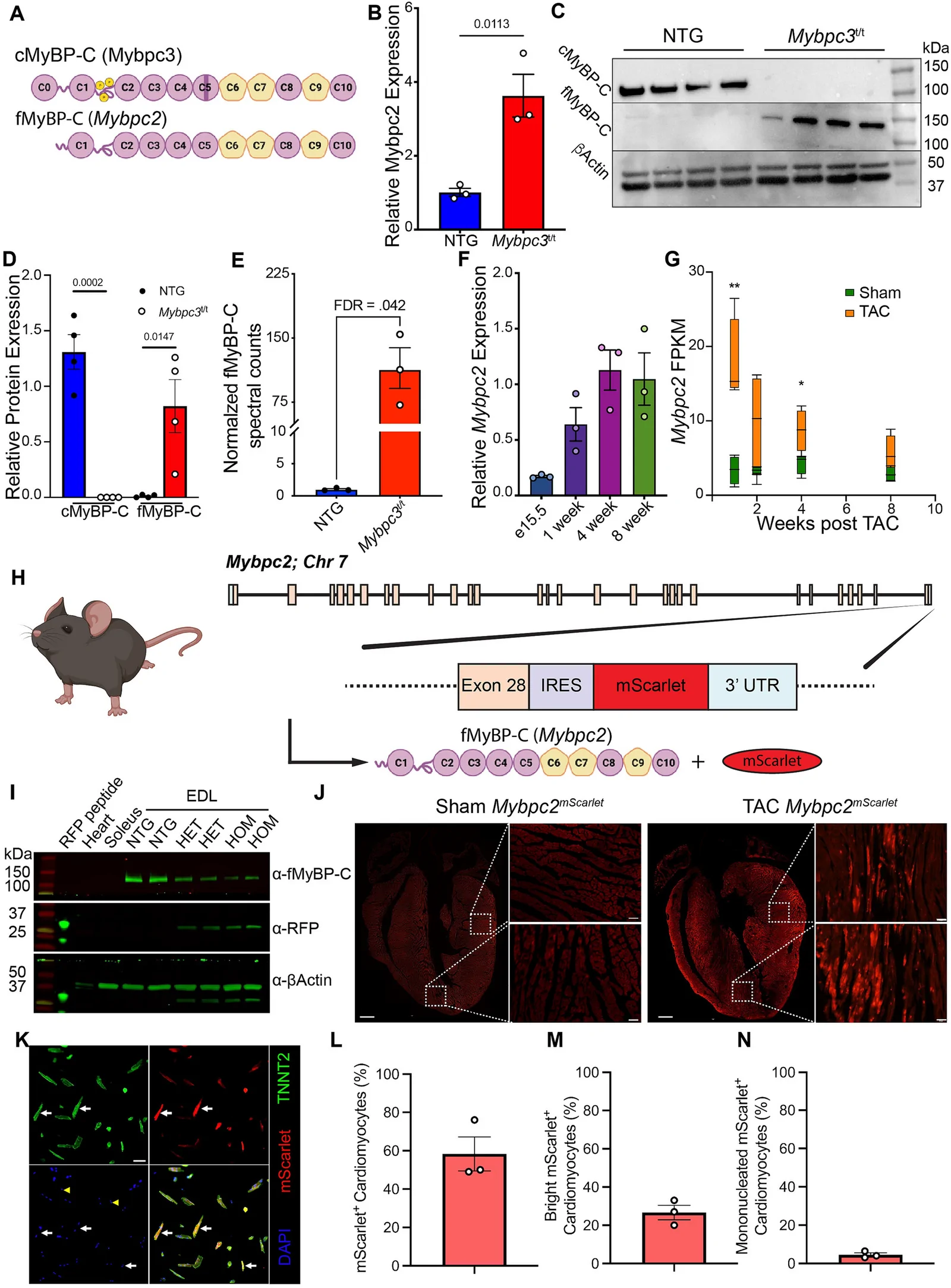
Upregulation of *Mybpc2* in response to cardiac stress. **A** Comparison of the domain structures of cMyBP-C and fMyBP-C. Circles represent immunoglobulin-like domains and pentagons fibronectin-like domains. Functionally annotated phosphorylation sites are indicated by “p,” and the cardiac-specific C5 insertion is demonstrated by the darkened rectangle. **B** Gene expression analysis of *Mybpc2* in non-transgenic (NTG) and *Mybpc3*^*t/t*^ mouse hearts (*n* = 3 per group). **C** and **D** Western blot analysis of cMyBP-C and fMyBP-C in NTG and *Mybpc3*^*t/t*^ mouse hearts (*n* = 4 per group). **E** Mass spectrometry spectral counts of fMyBP-C in NTG and *Mybpc3*^*t/t*^ ventricles (*n* = 3 per group). **F** *Mybpc2* expression in healthy mouse ventricle across developmental timepoints (n = 3 per group). **G** Transcriptional analysis of *Mybpc2* expression following TAC (data obtained from Geodata set GSE66630, *n* = 4 per group). **H** Gene targeting strategy for *Mybpc2*^mScarlet^ mice. **I** Western blot demonstrating specific expression of mScarlet to fast muscle type in naïve *Mybpc2*^mScarlet^ mice. **J** Representative immunofluorescent labeling of Sham and TAC *Mybpc2*^mScarlet^ mice, scale bar 500 μm and inset scale bar 200 μm. **K** Immunofluorescent labeling of enzymatically digested cardiomyocytes from *Mybpc2*^mScarlet^ mice following TAC, scale bar represents 100 μm. **L**–**N** Quantification of percent of mScarlet positive cardiomyocytes following TAC in *Mybpc2*.^mScarlet^ mice (*n* = 3). Data is expressed as mean ± SEM, except in F) in which minimum to maximum values are shown. *Represents *p* < 0.05, **p < 0.01, and ***p < 0.001

We and others have previously observed the upregulation of the skeletal sarcomeric gene *Mybpc2*, encoding fMyBP-C, in diseased hearts [4, 17, 45]. The myosin binding protein-C (MyBP-C) family are thick filament accessory proteins which regulate myocyte contractility via transient activation of thin filaments and inhibition of thick filaments (Fig. 1A) [11, 20, 25, 29, 30, 32]. fMyBP-C is almost exclusively expressed in fast-type muscles [40], while the adult heart expresses almost exclusively cardiac MyBP-C (cMyBP-C) [24]. Although these proteins share significant homology, there are key differences which may impart functional differences in the heart, including absence of regulatory phosphorylation sites and divergence of N-terminal domains [2, 24]. In vitro data from our previous studies has suggested fMyBP-C expression may induce cardiac dysfunction [18], but it is unknown whether its expression is a compensatory or pathological response in vivo.

To define the functional consequences of fMyBP-C expression in HF, we generated cardiac-specific *Mybpc2* transgenic mice (*Mybpc2*^Tg^). We observed mild hypertrophy and sarcomeric dysfunction in the absence of global cardiac dysfunction. This model was further crossed into a model of HF (*Mybpc3*^*t/t*^) and found that fMyBP-C expression exacerbated the cardiac phenotype. Conversely, adeno-associated viral-mediated silencing of *Mybpc2* partially restored cardiac function in *Mybpc3*^*t/t*^ mice. Finally, we performed transverse aortic constriction to induce HF in *Mybpc2* knockout (*Mybpc2*^KO^) mice. These mice exhibited greater resistance to HF. Overall, this study demonstrates that the upregulation of fMyBP-C in HF may be a maladaptive process which further exacerbates cardiac dysfunction.

## Materials and methods

An expanded Methods section is provided in the Online Data Supplement B.

All experiments were conducted under institutional guidelines approved by the University of Cincinnati (AM17-22–11-21–01) and the University of Arizona (2024–1236) Institutional Animal Care and Use Committee for Dr. Sadayappan and conform to the policies described in the Guide for the Use and Care of Laboratory Animals published by the National Institutes of Health. Mice were maintained on a 12 h light/dark cycle at ambient room temperature and provided access to water and standard chow ad libitum. Mice of both sexes were used across all studies, with balanced numbers. Sex was not considered a variable in any analysis. To generate transgenic mice with cardiac-specific expression of fMyBP-C, the fMyBP-C cDNA, *Mybpc2*, was amplified by polymerase chain reaction for cloning downstream of the alpha myosin heavy chain promoter. Briefly, an N-terminal cMyc tag for transgene recognition was cloned in-frame with *Mybpc2* and SalI restriction enzyme sites were included both 5’ and 3’ of the sequence for subcloning into the α-myosin heavy chain promoter (a gift from Dr. Jeffrey Robbins). Clones were screened for correct orientation of insertion and Sanger sequenced to confirm the *Mybpc2* sequence. The transgene was liberated from the vector backbone by digestion with NotI for zygote injections. Multiple *Mybpc2* transgenic (*Mybpc2*^Tg^) founders were identified, but only one showed appreciable expression of fMyBP-C in the heart. Global knockout models for *Mybpc2* [40] and *Mybpc3* [3, 23] have been previously described. A 5% isoflurane overdose by inhalation followed by cervical dislocation and bilateral thoracotomy was the method used for euthanasia of all mice. For quantitative polymerase chain reactions, total RNA was isolated from the hearts using the Qiagen RNeasy mini kit (74,104) following manufacturer’s procedures and cDNA synthesized using the iScript cDNA synthesis kit (BioRad). Proteins were isolated from ventricular muscle into either total or myofilament-enriched homogenates and blotted against target proteins using 4–15% Criterion tris–HCl acrylamide gels (BioRad). Cardiomyocytes were enzymatically isolated by Langendorff perfusion as previously described [20] and fixed with 4% paraformaldehyde for immunofluorescence imaging. Hearts used for histology (H&E or Masson’s Trichrome) or immunofluorescence were fixed with 4% paraformaldehyde, cryoprotected with sucrose, and embedded in OCT for sectioning. Hearts used for proteome-wide analyses were snap frozen and fractionated into cytoplasmic-, myofilament- and insoluble-enriched protein extracts as outlined [12] for analysis using an Orbitrap Fusion Lumos mass spectrometer. Cardiac function was measured non-invasively by echocardiography (anesthetized by 1–2% isoflurane inhalation) or terminally by intraventricular hemodynamic pressure measurement (anesthetized with ketamine-thiobutabarbital). Triton X-100 permeabilized ventricular fibers were used to assess force production and single nucleotide turnover as described [25].

All values are represented as mean ± SEM. Statistical analysis was performed using GraphPad Prism. Statistically significant differences between two groups were tested by an unpaired Student’s *t*-test. One-way or two-way ANOVA was used when comparing more than two groups, followed by post hoc analysis with Tukey’s multiple comparison test. All statistical significance was defined as *p* < 0.05.

## Results

### Upregulation of fMyBP-C in cardiac disease

To establish the framework for this study, we first validated our previous study, which demonstrated fMyBP-C expression is increased in models of cardiac disease [17]. In 8 week-old *Mybpc3*^t/t^ hearts, quantitative reverse transcriptase-PCR (qRT-PCR) revealed *Mybpc2* expression was increased nearly four-fold compared to non-transgenic (NTG) controls (Fig. 1B). Western blot analyses further demonstrated the elevated expression in fMyBP-C protein within diseased myocardium of *Mybpc3*^t/t^ mice (Fig. 1C, D). Mass spectrometry was used to validate this increase of the fMyBP-C protein quantity in the left ventricles of *Mybpc3*^t/t^ mice, demonstrating more than 100-fold induction (Fig. 1E). To determine whether this response was specific to *Mybpc3*^t/t^ mice, we interrogated transcriptomics datasets from additional *Mybpc3* mutant models, as well as *Tnnt2* and *Myh6* cardiomyopathy mouse models (Fig. S1A, B). Each of these cardiomyopathy models exhibited significantly increased *Mybpc2* expression, irrespective of *Mybpc3* expression. This suggests a pathogenic disease mechanism, rather than a compensatory transcriptional response to *Mybpc3* depletion. In the *Mybpc3* exon 33 deletion (*Mybpc3*^Δ33^) model of cardiomyopathy (Fig. S1C), *Mybpc2* was one of the top 5 most significantly increased genes compared to NTG littermates (Fig. S1D, E). Western blots showed that, similar to *Mybpc3*^t/t^ mice, fMyBP-C protein expression was significantly increased in *Mybpc3*^Δ33^ mice (Fig. S1F-H, [28]). By exploiting the cross-reactivity of this antibody, we estimated that fMyBP-C abundance in *Mybpc3*^Δ33^ hearts corresponded to approximately 10% of cMyBP-C levels in NTG mice (Fig. S1H).

Since re-expression of some skeletal muscle isoforms of muscle genes has previously been associated with fetal gene reprogramming in heart disease [46], we performed qRT-PCR on mouse hearts at e15.5, 1, 4, and 8-weeks old. Expression of *Mybpc2* mRNA was not elevated in fetal hearts (Fig. 1F), suggesting that this response may be distinct from the fetal gene program. We next investigated the expression of *Mybpc2* mRNA following transverse aortic constriction (TAC) surgery. Interrogation of publicly available RNA sequencing data [21] demonstrated the increased expression of *Mybpc2* in TAC hearts, which gradually decreased with time (Fig. 1G). To confirm this finding, we performed lineage tracing of *Mybpc2* using a reporter mouse (*Mybpc2*^*mScarlet*^) to express the fluorescent protein, mScarlet, under the control of the endogenous *Mybpc2* locus (Fig. 1H, I, Fig. S2). These mice were subjected to TAC surgery followed by histological assessment of reporter activation. Following TAC, *Mybpc2*^*mScarlet*^ hearts showed clear expression of mScarlet in hearts (Fig. 1J). A closer examination of enzymatically digested hearts confirmed the expression of mScarlet within most of the cardiomyocytes of *Mybpc2*^*mScarlet*^ mice following TAC surgery (Fig. 1K–N). Together, these data demonstrate that *Mybpc2* is aberrantly expressed in ventricular muscle and may associate with cardiac dysfunction in certain settings.

### Cardiac-specific expression of fMyBP-C induces subclinical cardiac dysfunction

**Fig. 2:**
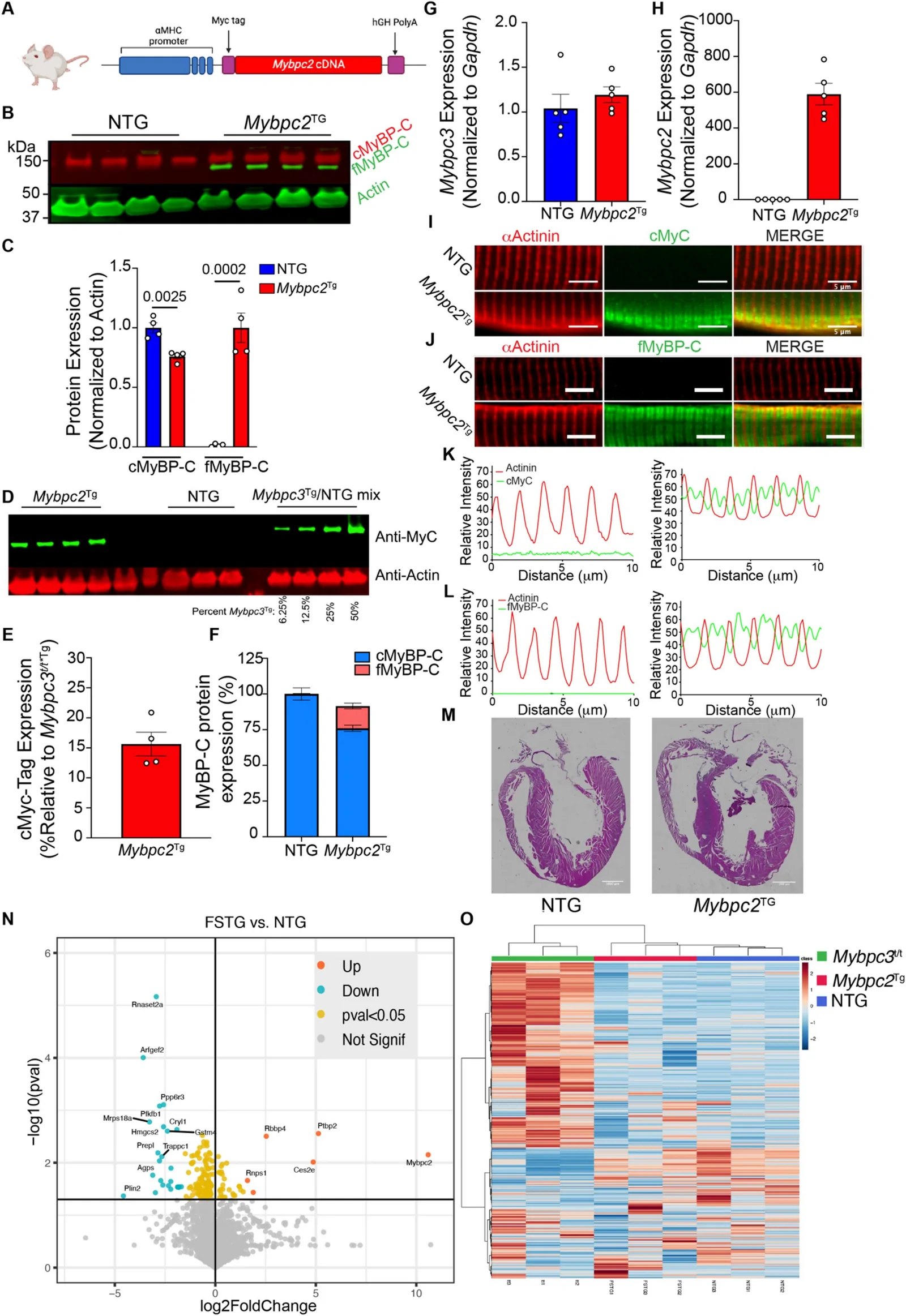
Generation of a mouse model to overexpress fMyBP-C in the heart. **A** Strategy for cardiac-specific over-expression of *Mybpc2*. **B** and **C** Western blot and quantification of cMyBP-C and fMyBP-C expression in NTG and *Mybpc2*^Tg^ mouse hearts (*n* = 4 per group). **D** and **E** Quantitative cMyc fusion tag expression Western blot for transgene expression against a standard curve to mouse hearts expressing 100% cMyc tagged cMyBP-C (*n* = 4). **F** Total MyBP-C expression levels determined by Western blot (*n* = 4). qRT-PCR analysis of **G** *Mybpc3* and **H** *Mybpc2* expression in in NTG and *Mybpc2*^Tg^ mouse hearts (*n* = 5 each). Immunofluorescent imaging of isolated cardiomyocytes stained for **I** α-actinin and cMyc-tag and **J** alpha-actinin and fMyBP-C, scale bar represents 5 μm. **K** and **L** Line scans corresponding to confocal images in **I** and **J**, respectively. **M** Representative histological section of NTG and *Mybpc2*^Tg^ mouse heart, scale bar represents 1 mm. **N** Volcano plot showing proteins that are differentially expressed between NTG and *Mybpc2*^Tg^ mouse heart as measured by mass spectrometry. **O** Heatmap showing hierarchical clustering of differentially expressed proteins between NTG, *Mybpc2*^Tg^, and *Mybpc3*^t/t^. Data is expressed as mean ± SEM. *P*-values are displayed within figure

To determine the consequence of increased fMyBP-C expression in the heart, we generated novel transgenic mice (Tg) to specifically overexpress *Mybpc2* in cardiomyocytes (*Mybpc2*^Tg^), driven by the *α*-myosin heavy chain promoter (Fig. 2A). Western blot analysis demonstrated the expression of fMyBP-C within the myofilament fraction of *Mybpc2*^Tg^ hearts (Fig. 2B), with a small, but significant decrease in cMyBP-C protein quantity (Fig. 2C). Importantly, the decrease in cMyBP-C protein was comparable to the increase in fMyBP-C, as determined by benchmarking myc tagged fMyBP-C expression against a standard curve generated using hearts that express 100% myc tagged cMyBP-C (Fig. 2D, E, Fig. S3). This finding critically demonstrates that total MyBP-C abundance is maintained in *Mybpc2*^Tg^ hearts. (Fig. 2F) and thus any functional changes are likely not due to reduced stoichiometry between myosin and MyBP-C. qRT-PCR established that while *Mybpc2* mRNA expression was highly increased in *Mybpc2*^Tg^ hearts, there was no change in *Mybpc3 levels* (Fig. 2G, H), suggesting that replacement of cMyBP-C by fMyBP-C occurs independently of changes in *Mybpc3* transcript abundance and is, therefore, likely mediated at the protein level. Further, fMyBP-C correctly localized to the A-bands of enzymatically digested *Mybpc2*^Tg^ cardiomyocytes, without disrupting cMyBP-C localization (Fig. 2I–L, Fig. S4). No major histopathology of *Mybpc2*^Tg^ hearts was observed, except for a mild dilatation of both ventricles (Fig. 2M). Mass spectrometry was performed on the ventricles of NTG and *Mybpc2*^Tg^ mice to assess changes in the proteome due to increased fMyBP-C protein quantity. Of the 3313 proteins quantified only 5% were modified (143 proteins were significantly reduced, 34 were increased); and the majority of these proteins were not significantly enriched in any gene ontology pathways (Fig. 2N). Indeed, only fatty acid metabolism was moderately enriched in downregulated proteins (Fold-enrichment 3.04, FDR = 4.51 × 10^−2^). Furthermore, hierarchical clustering of differentially expressed proteins between NTG, *Mybpc2*^Tg^, and *Mybpc3*^*t/t*^ further supported that the expression of fMyBP-C in otherwise healthy ventricles does not substantially impact the cardiac proteome (Fig. 2O).

**Fig. 3:**
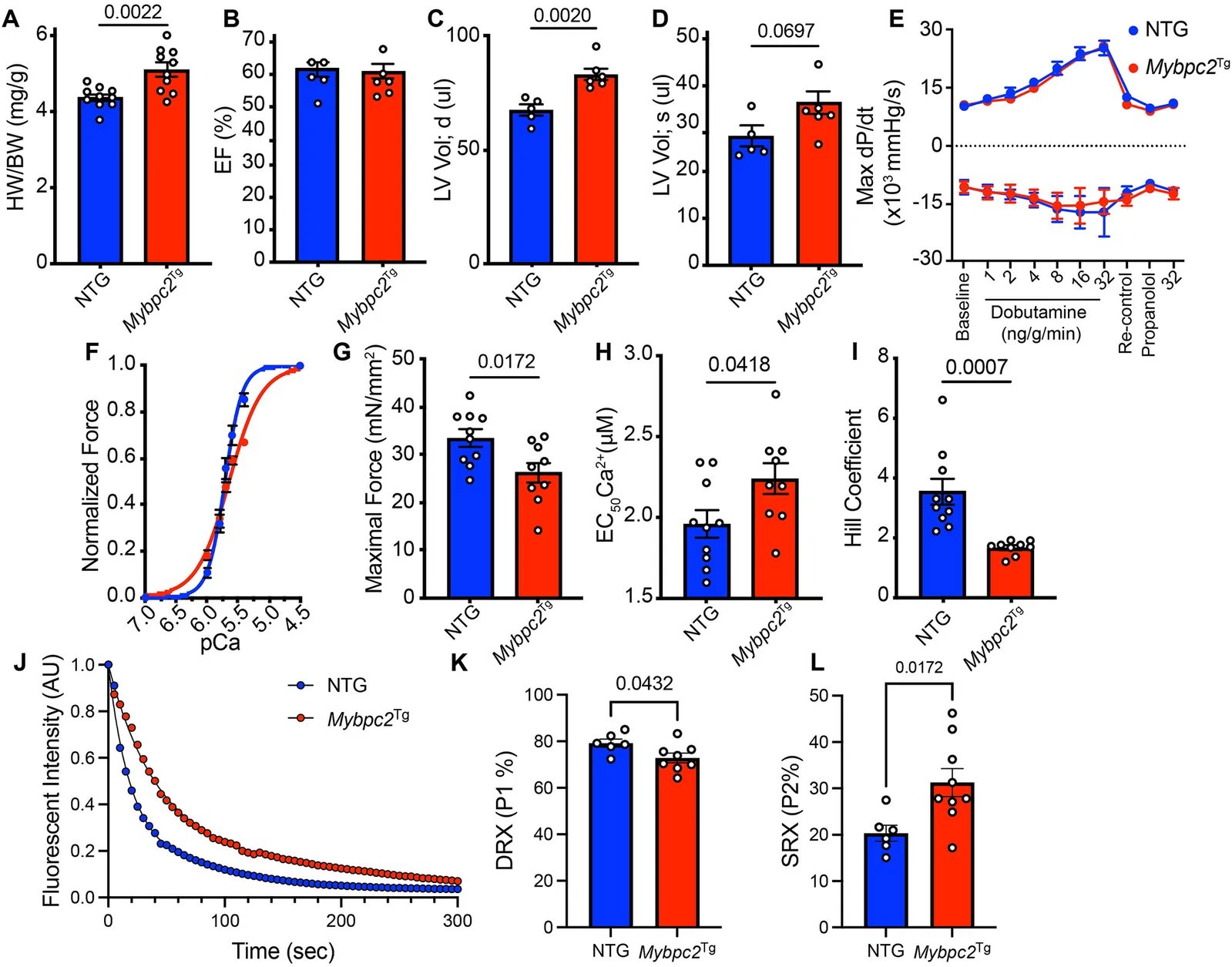
Dysregulation of sarcomere function due to expression of fMyBP-C in the heart. **A** 16-week-old heart weight to body weight ratio (*n* = 10 per group). 16-week-old **B** LV EF (%); **C** diastolic LV volume; and **D** systolic LV volume (*n* = 5 for NTG, 6 for *Mybpc2*^Tg^). **E** Intraventricular pressure catheterization with dobutamine stimulation (*n* = 4 for NTG, 5 for *Mybpc2*^Tg^). **F** Normalized force production of skinned papillary muscles (*n* = 10 fibers for NTG, 9 for *Mybpc2*^Tg^ from 3–4 mice). **G** Maximal force production; **H** calcium sensitivity; and **I** Hill Coefficient of skinned papillary muscles. **J** mantATP nucleotide turnover assay (*n* = 6 for NTG and 9 fibers from 3 mice for NTG and *Mybpc2*^Tg^, respectively). **K** Percentage of DRX and **L** SRX myosin heads. Data is expressed as mean ± SEM. *P*-values are displayed within the figure

We next defined the functional consequences of fMyBP-C expression in the heart by assessing cardiac function via echocardiography (Supplementary Table 1) and invasive hemodynamics in 12-week-old mice. Heart weight-to-body weight ratio (Fig. 3A) was significantly increased in *Mybpc2*^Tg^ mice, in the absence of changes in ejection fraction (Fig. 3B). Echocardiography also demonstrated increased cardiac dimensions during both systole and diastole (Fig. 3C, D), while in vivo pressure measurements were unchanged at baseline and with dobutamine challenge (Fig. 3E). Together, these data demonstrate that fMyBP-C expression in the heart promotes mild ventricular enlargement in the absence of functional changes at the organ level.

Skinned papillary muscle fibers were used for force-pCa mechanics to assess potential subclinical deficits in contractile function (Fig. 3F). *Mybpc2*^Tg^ muscle fibers produced significantly less active force than NTG and exhibited reduced myofilament calcium sensitivity (Fig. 3G, H). Notably, *Mybpc2*^Tg^ muscle fibers exhibited a lower Hill constant (Fig. 3I), suggesting the co-operativity of muscle activation is blunted upon expression of fMyBP-C. We also observed that skinned *Mybpc2*^Tg^ muscles exhibit greater stability of the myosin super-relaxed (SRX) state (Fig. 3J–L). It is unclear if this is due to myosin binding differences between fMyBP-C and cMyBP-C or a total reduction in MyBP-C phosphorylation, which is known to stabilize the SRX [13, 25]. Collectively, these data suggest that low level expression of fMyBP-C in the heart is sufficient to drive subclinical deficits in myofilament contractility. Mechanistically, these findings suggest that fMyBP-C promotes stabilization of myosin heads within the SRX state, thereby reducing myosin availability for force generation and impairing muscle activation.

### Exclusive expression of fMyBP-C exacerbates ventricular dysfunction

**Fig. 4:**
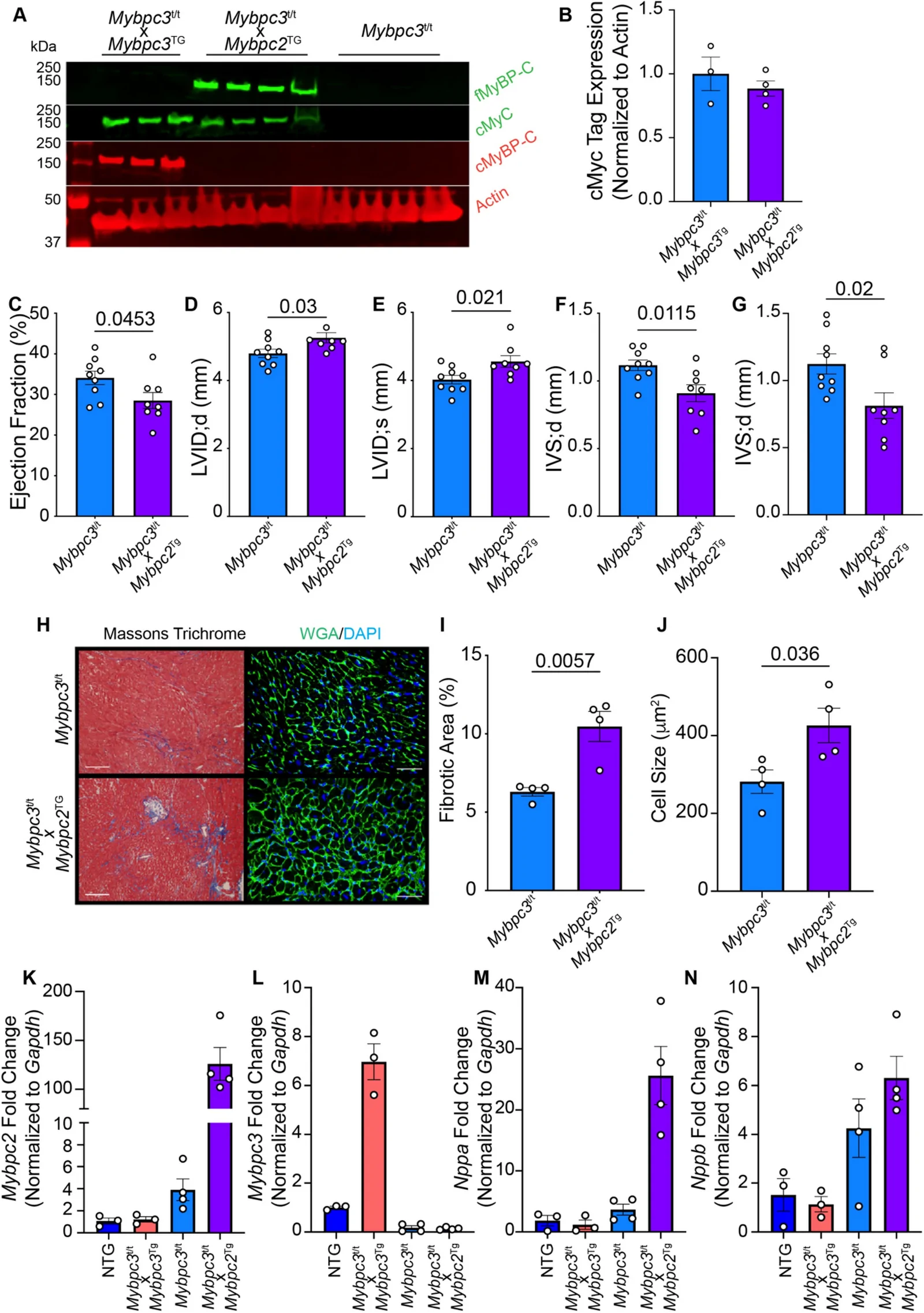
Exclusive expression of cMyBP-C with fMyBP-C exacerbates cardiac pathology. **A** Western blot to assess fMyBP-C protein levels in *Mybpc3*^*t/t*^* xMybpc2*^Tg^ mouse hearts. **B** Quantification of cMyc tag expression between *Mybpc3*^*t/t*^* xMybpc3*^Tg^ (*n* = 3) and *Mybpc3*^*t/t*^* xMybpc2*^Tg^ (*n* = 4). **C**–**G** Echocardiographic parameters (*n* = 9 for *Mybpc3*^*t/t*^; 8 for *Mybpc3*^*t/t*^* xMybpc2*^Tg^). **H** Masson’s trichrome (scale bar = 100 μm) and wheat germ agglutinin (scale bar = 50 μm). **I** Fibrosis and **J** cell size quantification (*n* = 4 per group). **K**–**N** qRT-PCR expression of *Mybpc2, Mybpc3*, *Nppa,* and *Nppb* (*n* = 3–4 per group). Data is expressed as mean ± SEM. *P*-values are displayed within figure

Having demonstrated that incorporation of fMyBP-C into the cardiac sarcomere causes mild dysfunction, we investigated the consequence of 100% expression of fMyBP-C in the mouse heart. To achieve this, we crossed the *Mybpc2*^Tg^ with a cMyBP-C null mouse model (*Mybpc3*^t/t^). Western blots demonstrated that fMyBP-C expression in *Mybpc3*^t/t^x*Mybpc2*^Tg^ mice was comparable to cMyBP-C expression in *Mybpc3*^t/t^x*Mybpc3*^Tg^ mice, a rescue model known to express near-normal levels of cMyBP-C (Fig. 4A, B, [38]). Echocardiography was performed on 12-week-old mice (Supplementary Table 2), which demonstrated that exclusive fMyBP-C overexpression was sufficient to exacerbate cardiac dysfunction in this established cardiomyopathy model (Fig. 4C). Similar to *Mybpc2*^*Tg*^ mice, the left ventricular chamber dimension in *Mybpc3*^t/t^x*Mybpc2*^Tg^ mice was enlarged (Fig. 4D, E), while interventricular septal thickness was significantly reduced (Fig. 4F, G). Histological analyses revealed greater fibrosis (Fig. 4H, I) in the *Mybpc3*^t/t^x*Mybpc2*^Tg^ model compared to *Mybpc3*^t/t^ mice, as well as increased cardiomyocyte hypertrophy (Fig. 4H, J). While *Mybpc2* expression was still fourfold elevated in *Mybpc3*^t/t^ mice compared to NTG, this was more prominent in *Mybpc3*^t/t^x*Mybpc2*^Tg^ mice (Fig. 4K). Meanwhile, *Mybpc3* expression was reduced in both *Mybpc3*^t/t^ and *Mybpc3*^t/t^x*Mybpc2*^Tg^ mice (Fig. 4L). Furthermore, we also observed elevated levels of *Nppa* only in *Mybpc3*^t/t^x*Mybpc2*^Tg^ mice (Fig. 4M), while *Nppb* was higher in both *Mybpc3*^t/t^ and *Mybpc3*^t/t^x*Mybpc2*^Tg^ mice compared to controls (Fig. 4N). This data suggests, somewhat counterintuitively, that the exclusive expression of fMyBP-C is more detrimental than the complete absence of cMyBP-C with respect to cardiac function.

**Fig. 5:**
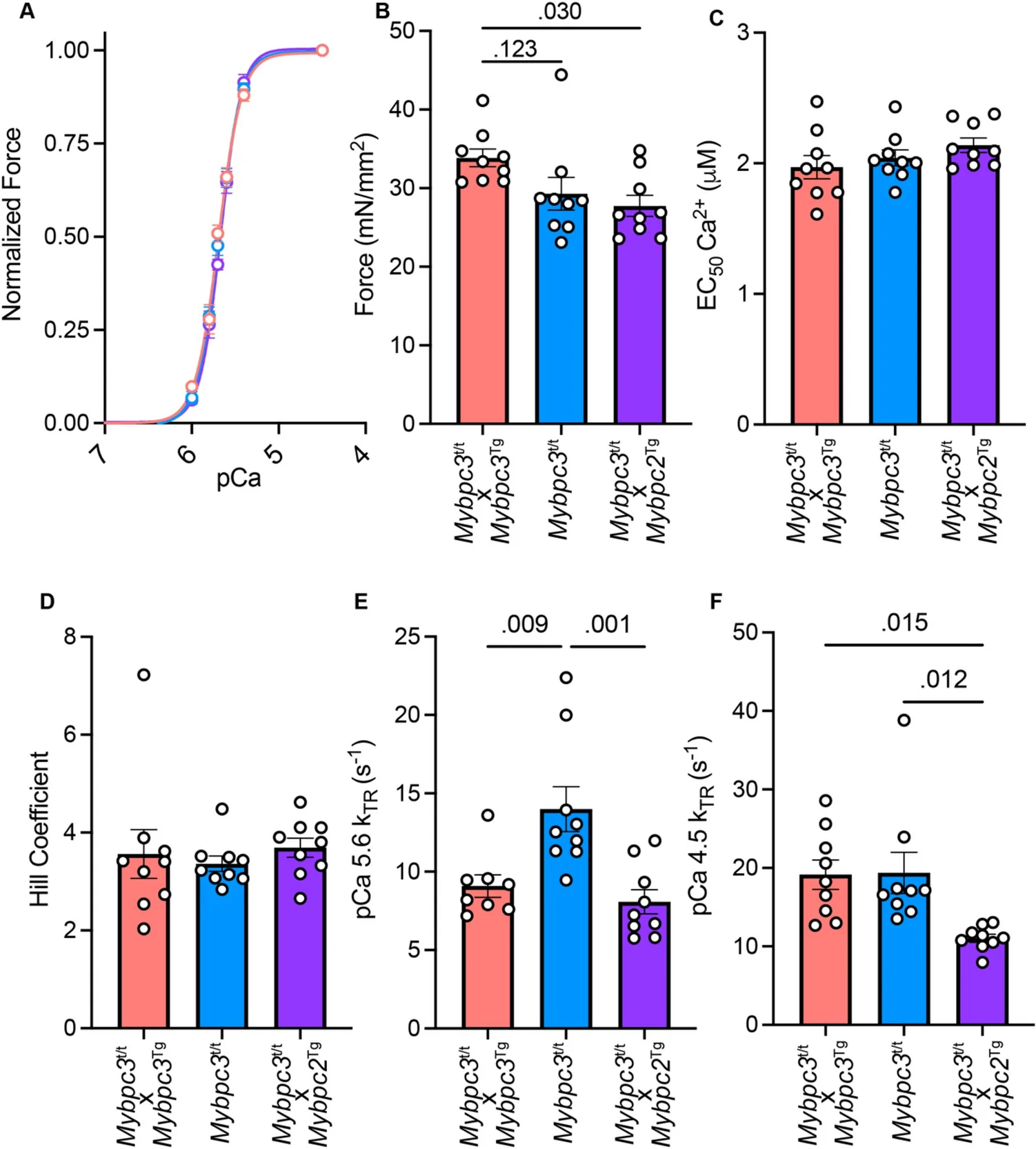
Impaired force and kinetics in cardiac muscle with complete fMyBP-C replacement. **A** Normalized force production of skinned papillary muscles. **B** Maximal force production; **C** calcium sensitivity; and **D** Hill Coefficient from skinned papillary muscles. k_TR_ measured at **E** intermediate and **F** maximal calcium concentration (*n* = 8–9 fibers per group, 3 mice per group). Data is expressed as mean ± SEM. *P*-values are displayed within the figure

To further understand how complete expression of fMyBP-C affects heart function, skinned ventricular fibers from *Mybpc3*^t/t^x*Mybpc2*^Tg^ were compared to *Mybpc3*^t/t^ and *Mybpc3*^t/t^x*Mybpc3*^Tg^ fibers in force-pCa experiments (Fig. 5A). Maximal force production (Fig. 5B) was significantly reduced in *Mybpc3*^t/t^x*Mybpc2*^Tg^ mice compared to control *Mybpc3*^t/t^x*Mybpc3*^Tg^ mice. No differences were noted in calcium sensitivity (Fig. 5C) or Hill slope (Fig. 5D) between groups. To assess the rate of contraction, rapid slack re-stretch assays were performed at intermediate and maximal calcium concentrations. In line with established results [14], *Mybpc3*^*t/t*^ mice exhibited accelerated tension redevelopment (*k*_TR_) at submaximal calcium activation (Fig. 5E), but comparable to control fibers at maximal calcium levels (Fig. 5F). Conversely, the submaximal *k*_TR_ of *Mybpc3*^t/t^x*Mybpc3*^Tg^ preparations were comparable to control mice (Fig. 5E), but could not normalize at maximal levels (Fig. 5F). This data collectively highlights key functional differences in the cardiac and fast skeletal paralogs of MyBP-C which may differentially impact cellular contractility and emphasizes the inability of fMyBP-C to functionally replace its cardiac paralog.

### AAV-mediated *Mybpc2 *silencing partially ameliorates HF* in Mybpc3*^*t/t*^ mice

**Fig. 6:**
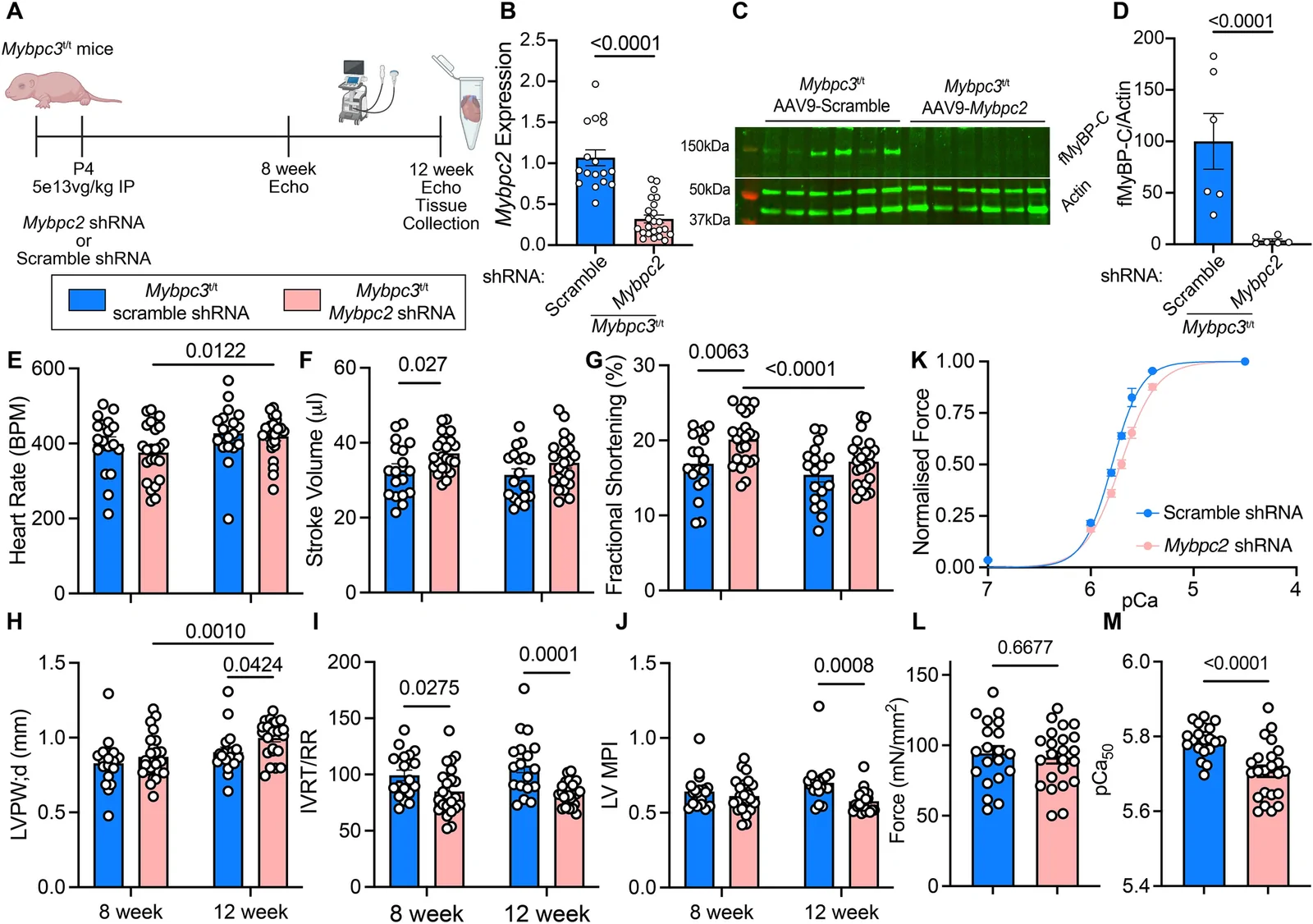
*Mybpc2* silencing partially restores cardiac function in *Mybpc3*^t/t^ mice. **A** Schematic of AAV9-shRNA experiment in *Mybpc3*^t/t^ mice. **B** qRT-PCR of *Mybpc2* expression in LV tissue (*n* = 17 and 23 for AAV9-scramble and AAV9-*Mybpc2*, respectively. Welch’s *t*-test). **C** Western blot against fMyBP-C and actin between AAV9-scramble and AAV9-*Mybpc2* **D** Quantification of Western blot data (*n* = 6 per group. Lognormal Welch’s *t*-test). **E** Heart rate, **F** stroke volume, **G** fractional shortening, **H** diastolic left ventricular wall thickness, **I** Heart rate normalized isovolumic relaxation time, and **J** left ventricular myocardial performance index parameters from echocardiography (n = 18 and 23 for AAV9-scramble and AAV9-*Mybpc2*, respectively. Two-way ANOVA with repeated measures). **K** Force-pCa curve from detergent skinned LV muscle (*n* = 21 and 23 for AAV9-scramble and AAV9-*Mybpc2*, respectively). **L** Maximal force production, and **M** calcium sensitivity (Welch’s *t*-test). Data are presented as mean ± SEM. *P*-values are displayed within figure

As overexpression of *Mybpc2* accelerates cardiac dysfunction in *Mybpc3*^t/t^ mice lacking cMyBP-C, we next tested whether reducing *Mybpc2* expression could provide therapeutic benefit. To test this, we treated neonatal (P4) *Mybpc3*^t/t^ mice with 5e13vg/kg of either AAV9-*Mybpc2* shRNA or AAV9-Scramble shRNA (VectorBuilder) via intraperitoneal injection (Fig. 6A). AAV9-*Mybpc2* shRNA reduced *Mybpc2* expression by 70% in ventricular tissue (Fig. 6B), which corresponded to 95% reduction in fMyBP-C protein abundance compared to controls (Fig. 6C, D). We performed echocardiography at 8 and 12-weeks of age to assess in a blinded manner whether *Mybpc2* silencing imparted any functional benefit. In the absence of any changes in heart rate (Fig. 6E), mice treated with AAV9-*Mybpc2* shRNA exhibited a significant increase in both stroke volume (Fig. 6F) and fractional shortening (Fig. 6G) at 8 weeks of age. While these values remained higher at 12 weeks, this did not reach significance. Ventricular posterior wall thickness was modestly increased in AAV9-*Mybpc2*-shRNA-treated mice, consistent with partial normalization of cardiac geometry (Fig. 6H). Furthermore, diastolic measurement of cardiac function revealed improvements in the isovolumic relaxation time (Fig. 6I) as well as myocardial performance index at 12 weeks (Fig. 6J). Using chemically skinned myocardial preparations to assess force-pCa relationships (Fig. 6K), we found that *Mybpc2* silencing did not alter maximal force generation (Fig. 6L). However, AAV9-*Mybpc2* shRNA treated myocardium exhibited a small, but reproducible reduction in myofilament calcium sensitivity (ΔpCa = − 0.1), corresponding to a rightward shift in the force-pCa relationship (Fig. 6M). Taken together, these findings demonstrate that disease-associated induction of *Mybpc2* contributes to cardiac dysfunction in *Mybpc3* cardiomyopathy and identify *Mybpc2* as a potential therapeutic target.

### Deletion of *Mybpc2* diminishes pressure overload-induced HF

**Fig. 7:**
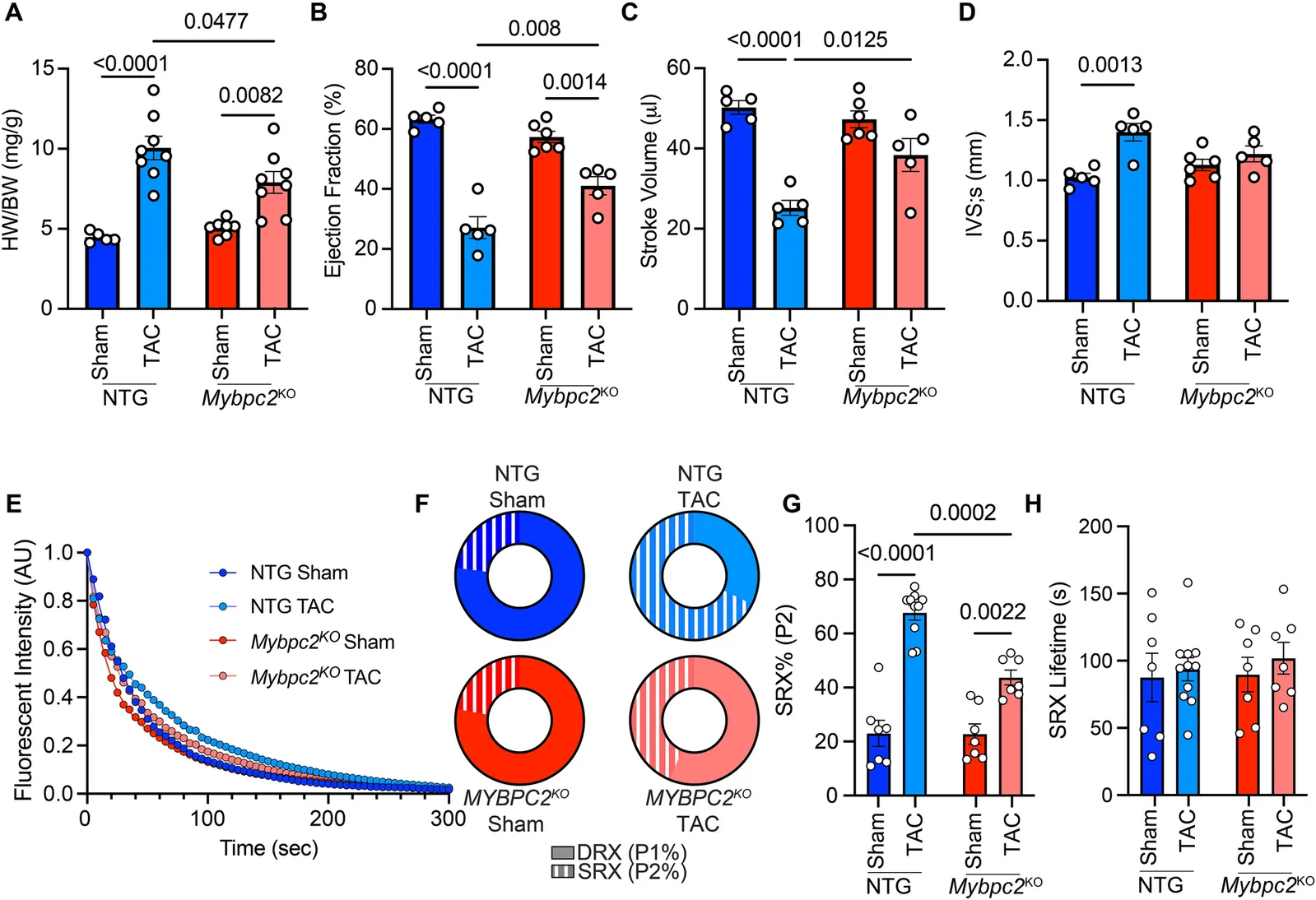
Deletion of *Mybpc2* ameliorates dysfunction in response to pressure overload. **A** Heart to body weight ratios (*n* = 5, 8, 7, 8 for NTG Sham, NTG TAC, *Mybpc2*^*KO*^ Sham*,* and *Mybpc2*^*KO*^ TAC, respectively)*.* **B**–**D** Echocardiographic parameters of NTG and *Mybpc2*^−/−^ sham and TAC mice (n = 5, 5, 6, 5 per group as above)**. E** mantATP nucleotide turnover assay experiments from skinned ventricular muscle from NTG and *Mybpc2*^*KO*^ Sham and TAC mice. **F** Relative proportions of DRX and SRX myosin between groups expressed as parts of a whole. **G** SRX populations and **H** SRX lifetimes from single myosin ATP turnover experiments (n = 10–13 fibers per group, *n* = 3 mice). Statistical tests are two-way ANOVA with Tukey post hoc test. Data is expressed as mean ± SEM. *P*-values are presented within the figure

Having established that *Mybpc2* silencing partially rescues cardiac dysfunction in *Mybpc3*^t/t^ mice, we next asked whether loss of *Mybpc2* could also protect against other forms of HF. Since *Mybpc2* was found to be upregulated in the setting of pressure overload (Fig. 1G, J), we chose this model to test this hypothesis. Eight-week-old NTG or *Myb*pc2^KO^ mice [40] were subjected to transverse aortic constriction (TAC) for 8 weeks to induce pressure overload-induced HF. While heart weight to body weight ratio was significantly elevated following TAC in both NTG and *Myb*pc2^KO^ mice compared to sham mice, this was ameliorated in *Myb*pc2^KO^ mice (Fig. 7A). Similarly, *Myb*pc2^KO^ mice exhibited greater protection from impaired contractile function (ejection fraction and stroke volume, Fig. 7B, C) and wall thickening (Fig. 7D) compared to NTG mice. Strikingly, single ATP turnover assays (Fig. 7E) in skinned ventricular fibers revealed a stabilized population of SRX myosin in NTG mice following TAC compared to Sham mice (Fig. 7F–H). While deletion of *Mybpc2* had no impact on SRX at baseline, the TAC-induced stabilization of SRX myosin was attenuated in *Mybpc2*^KO^ mice relative to NTG mice (Fig. 7F–H). Collectively, this data demonstrates that the genetic deletion of *Mybpc2* prior to pressure overload-induced HF mitigates cardiac dysfunction. Importantly, these findings identify pathological stabilization of SRX myosin as a feature of pressure-overload HF that is partially corrected by deletion of *Mybpc2*.

## Discussion

Our study demonstrates that disease-associated induction of the fast skeletal myosin binding protein-C paralog, *Mybpc2* or fMyBP-C, is maladaptive in the heart. Across multiple models of cardiac stress, including genetic cardiomyopathy and pressure overload, *Mybpc2* expression was increased within ventricular myocardium. In contrast, our previous studies showed that *Myb*pc2^KO^ mice exhibited no abnormal cardiac function [40]. While the functional significance of this response was unknown, we show that fMyBP-C impairs contractile performance, exacerbates disease progression, and alters myosin SRX. Conversely, viral-mediated silencing or genetic ablation partially improved cardiac function in both genetic and pressure overload models of HF. Together, these findings identify *Mybpc2* as a previously unrecognized contributor to cardiac dysfunction and suggest that suppression of *Mybpc2* may represent a novel therapeutic strategy.

Three *Mybpc* genes are expressed in mammals: *Mybpc1* (slow skeletal), *Mybpc2* (fast skeletal), and *Mybpc3* (cardiac). Although these encode proteins with substantial structural homology (Fig. 1A, [18]), it has long been recognized that the skeletal and cardiac paralogs do not exhibit functional transcomplementation in the healthy heart [6, 8]. However, our group has recently demonstrated that ectopic expression of *Mybpc2*, the gene encoding the fast paralog of MyBP-C, is upregulated in the hearts of mice lacking *Mybpc3* [17]*.* Importantly, this response was not restricted to *Mybpc3* deficiency, but was also observed in multiple independent cardiomyopathy models and following pressure overload. These findings suggest that *Mybpc2* induction represents a broader response to cardiac stress rather than a compensatory consequence of *Mybpc3* depletion alone.

A key question arising from these observations is why *Mybpc2* expression is induced in disease hearts. One possibility was that fMyBP-C forms part of the fetal gene program, whereby developmental genes are reactivated in response to pathological stress [42]. Several sarcomeric proteins follow this behavior including *Myh7* and the fetal N2BA isoform of *TTN* [19]. Several skeletal muscle genes have similarly been implicated in this adaptive response [46]. However, we found no evidence that *Mybpc2* is expressed during fetal cardiac development, suggesting that its induction in disease is mechanistically distinct from classical fetal gene reprogramming. Instead, we speculate that *Mybpc2* expression may represent an attempt to adapt to altered mechanical demands placed on the diseased myocardium.

In skeletal muscle, fMyBP-C is preferentially expressed in fast glycolytic myofibers [40], which are recruited during high-load activities and generate greater peak force than oxidative myofibers. Previous studies suggest that fMyBP-C contributes to this functional specialization by modulating actomyosin interactions and slowing actin filament sliding, thereby promoting cooperative recruitment of force-generating crossbridges [15]. It is, therefore, conceivable that fMyBP-C initially serves as a compensatory response to ventricular wall stress. However, our data demonstrate that any initial benefit is outweighed by detrimental effects on cardiac performance.

Several structural and regulatory differences between fast skeletal and cardiac paralogs may also underlie these effects. Unlike cMyBP-C, fMyBP-C lacks the cardiac-specific N-terminal C0 domain (Fig. 1A), which contributes to both thick and thin filament regulation [34, 36]. Furthermore, the major phosphorylation sites that mediate adrenergic regulation of cMyBP-C are absent from fMyBP-C. As cMyBP-C phosphorylation is critical for normal cardiac function [37], loss of these regulatory sites in fMyBP-C is likely to alter sarcomere behavior. Consistent with this concept, we observed reduced force generation and impaired tension redevelopment kinetics in mice expressing fMyBP-C. Similarly, it was shown that adenoviral overexpression of fMyBP-C appeared to affect cellular contractility in enzymatically isolated adult rat cardiomyocytes [18]. Counterintuitively, we observed a small, but significant reduction in calcium sensitivity in *Mybpc3*^t/t^ mice treated with an *Mybpc2* shRNA. Additional studies are required to determine the mechanism underlying this observation.

Our findings further suggest that altered regulation of the myosin super-relaxed state (SRX) may contribute to these contractile phenotypes. Expression of fMyBP-C increased SRX stability, whereas deletion of *Mybpc2* attenuated pathological SRX stabilization following pressure overload. While the biochemical SRX does not perfectly correspond to the structural “OFF” state of myosin, increased SRX occupancy would be predicted to reduce the number of myosin heads available for force production, and thereby slow contractile activation. We, therefore, propose that fMyBP-C alters thick filament regulation, at least in part, through modulation of myosin state transitions. Whether this arises directly from differences in myosin binding, altered thin filament activation, or a combination of both mechanisms remains to be determined. Future structural studies using X-ray diffraction or cryo-electron microscopy will be required to resolve these possibilities.

In conclusion, we have demonstrated that induction of fMyBP-C is a maladaptive feature of cardiac stress. Expression of fMyBP-C impairs contractile performance and exacerbates disease progression, whereas ablation of fMyBP-C expression improves cardiac function in HF models. These findings identify fMyBP-C as a novel regulator of cardiac contractility in cardiac disease and suggest that the therapeutic suppression of *Mybpc2* may represent a potential strategy for the treatment of certain forms of HF.

## Supplementary Information

Below is the link to the electronic supplementary material.

Supplementary file1 (PDF 997 KB)

Supplementary file2 (PDF 200 KB)

## Data Availability

The mass spectrometry proteomics data have been deposited to the ProteomeXchange Consortium via the PRIDE partner repository with the dataset identifier PXD047728. RNA sequencing data has been deposited in the NCBI Gene Expression Omnibus. *Mybpc3*^t/t^ (GSE335239); *Mybpc3*^Δ33^ (GSE335235). All datasets will be made publicly accessible following publication.

## Abbreviations

HF: Heart failure
*Mybpc2*: Mouse fast myosin binding protein-C gene
*Mybpc3*: Mouse cardiac myosin binding protein-C gene
cMyBP-C: Cardiac myosin binding protein-C
fMyBP-C: Fast myosin binding protein-C
*Mybpc2*^Tg^: α-Myosin heavy chain-driven mouse fast myosin binding protein-C transgene
*Mybpc3*^*t/t*^: Mouse cardiac myosin binding protein-C null
*Mybpc2*^KO^: Mouse fast myosin binding protein-C knockout
NTG: Non-transgenic mice (wild type)
TAC: Transverse aortic constriction
Tg: Transgenic mice
SRX: Super-relaxed state of myosin
