# Supplementary material for: Fast skeletal myosin binding protein-C expression exacerbates dysfunction in heart failure
Source: Basic Res Cardiol. 2026 Aug 5;121(5):1047–61. doi: 10.1007/s00395-026-01202-8 (PMC13593655; doi:10.1007/s00395-026-01202-8)
Supplement: Supplementary file 1 [file 395_2026_1202_MOESM1_ESM.pdf]

Appendix A: Online Supplementary Data

Fast skeletal myosin binding protein-C expression exacerbates dysfunction in heart failure

James W. McNamara[1,2,3*], Taejeong Song[1,4], Perwez Alam[5,#], Akhil Baby[1,4,6], Aleksandra Binek[7], Kalyani Ananthamohan[1,4], Rohit R. Singh[1§], Michelle L. Nieman[8], Marloes van den Berg[4], Brent M. Cernyar[4], Sheryl E. Koch[1], Malina J. Ivey[5], Thomas L. Lynch IV[9], James T. Pearson[10], Jack Rubinstein[1], J-P Jin[11€], John N. Lorenz[8], Jennifer E. Van Eyk[7,12], Onur Kanisicak[5†] and Sakthivel Sadayappan[1,4*]

[1]Center for Cardiovascular Research, Division of Cardiovascular Health and Disease, Department of Internal Medicine, University of Cincinnati, Cincinnati, OH, USA

[2]Murdoch Children's Research Institute, The Royal Children's Hospital, Parkville, Victoria 3052; Novo Nordisk Foundation Centre for Stem Cell Medicine (reNEW), Murdoch Children's Research Institute, Melbourne, Victoria, Australia

[3]Victor Chang Cardiac Research Institute, Victor Chang Cardiac Research Institute, Darlinghurst, New South Wales, Australia

[4]Department of Cellular and Molecular Medicine, Sarver Heart Center, University of Arizona College of Medicine, Tucson, Arizona, USA

[5]Department of Pathology and Laboratory Medicine, University of Cincinnati College of Medicine, Cincinnati, OH, USA.

[6]Department of Genetic Engineering, School of Biotechnology, Madurai Kamaraj University, Madurai, India

[7]Advanced Clinical Biosystems Research Institute, Smidt Heart Institute, Cedars-Sinai Medical Center, Los Angeles, California 90048, USA.

[8]Department of Pharmacology and Systems Physiology, University of Cincinnati College of Medicine, Cincinnati, OH, USA

[9]Department of Cell and Molecular Physiology, Loyola University Chicago, Maywood, IL, USA

[10]Department of Cardiac Physiology, National Cerebral and Cardiovascular Center Research Institute, Suita-shi, Osaka, Japan.

[11]Department of Physiology, Wayne State University School of Medicine, Detroit, MI, USA.

[12]Department of Biomedical Sciences, Cedars-Sinai Medical Center, Los Angeles, California 90048, USA.

[§]<u>Current Address</u>: Amgen, Discovery Protein Science, South San Francisco, CA 94080.

[#]<u>Current Address</u>: Department of Biomedical Sciences, University of Missouri, Columbia, Missouri-65211, USA.

[€]<u>Current Address</u>: Department of Phyiology and Biophysics, University of Illinois at Chicago, 835 S Wolcott Ave., Chicago, IL 60612.

* To whom correspondence should be addressed: j.mcnamara@victorchang.edu.au, sadayappan@arizona.edu

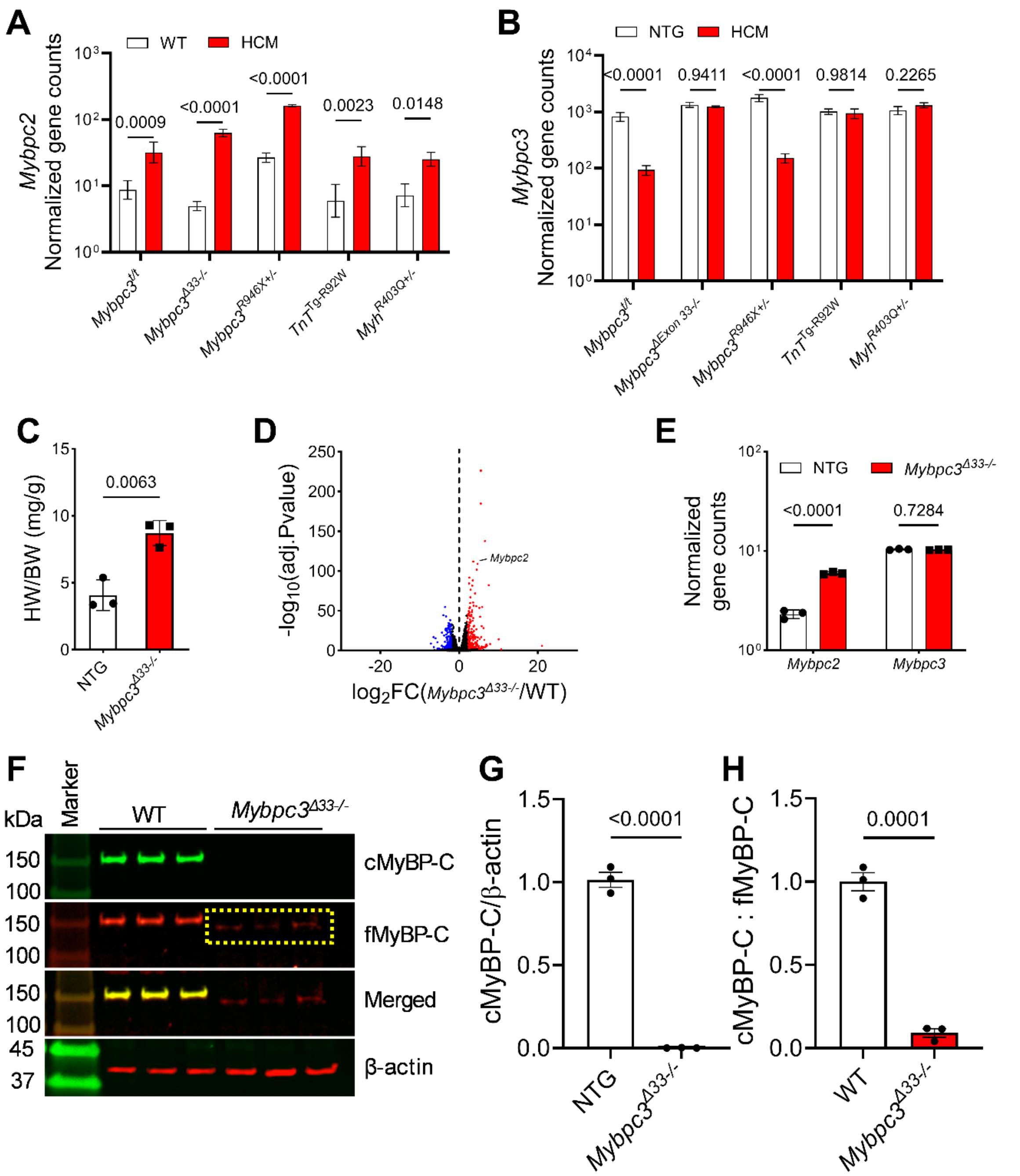

Supplementary Figure 1. Increased *Mybpc2* expression across multiple murine models of HCM models. A. *Mybpc2* and B. *Mybpc3* gene expression across multiple murine models of HCM demonstrate increased *Mybpc2* expression. C. Increased heart weight to body weight in *Mybpc3*$^{\Delta 33\text{-/-}}$ mice. D. Volcano plot of differentially expressed genes demonstrates increased *Mybpc2* gene expression. E. Relative expression of *Mybpc2* and *Mybpc3* between WT and *Mybpc3* exon 33 mice. F. Western blot of WT and *Mybpc3* exon 33 hearts demonstrating increased fMyBP-C expression in *Mybpc3*$^{\Delta 33\text{-/-}}$ hearts. G. Quantification of cMyBP-C protein expression. H. Relative quantification of fMyBP-C in *Mybpc3*$^{\Delta 33\text{-/-}}$ hearts compared to cMyBP-C expression in WT hearts, using cross-reactivity of fMyBP-C antibody. Statistical significance was determined by two-way ANOVA with Šídák's multiple comparisons test (Panel. A, B and E) or unpaired Student's t-test (Fig. C, G and H). **P < 0.01, ***P < 0.001, ****P < 0.0001. Data are presented as mean ± SEM, n = 3 per group.

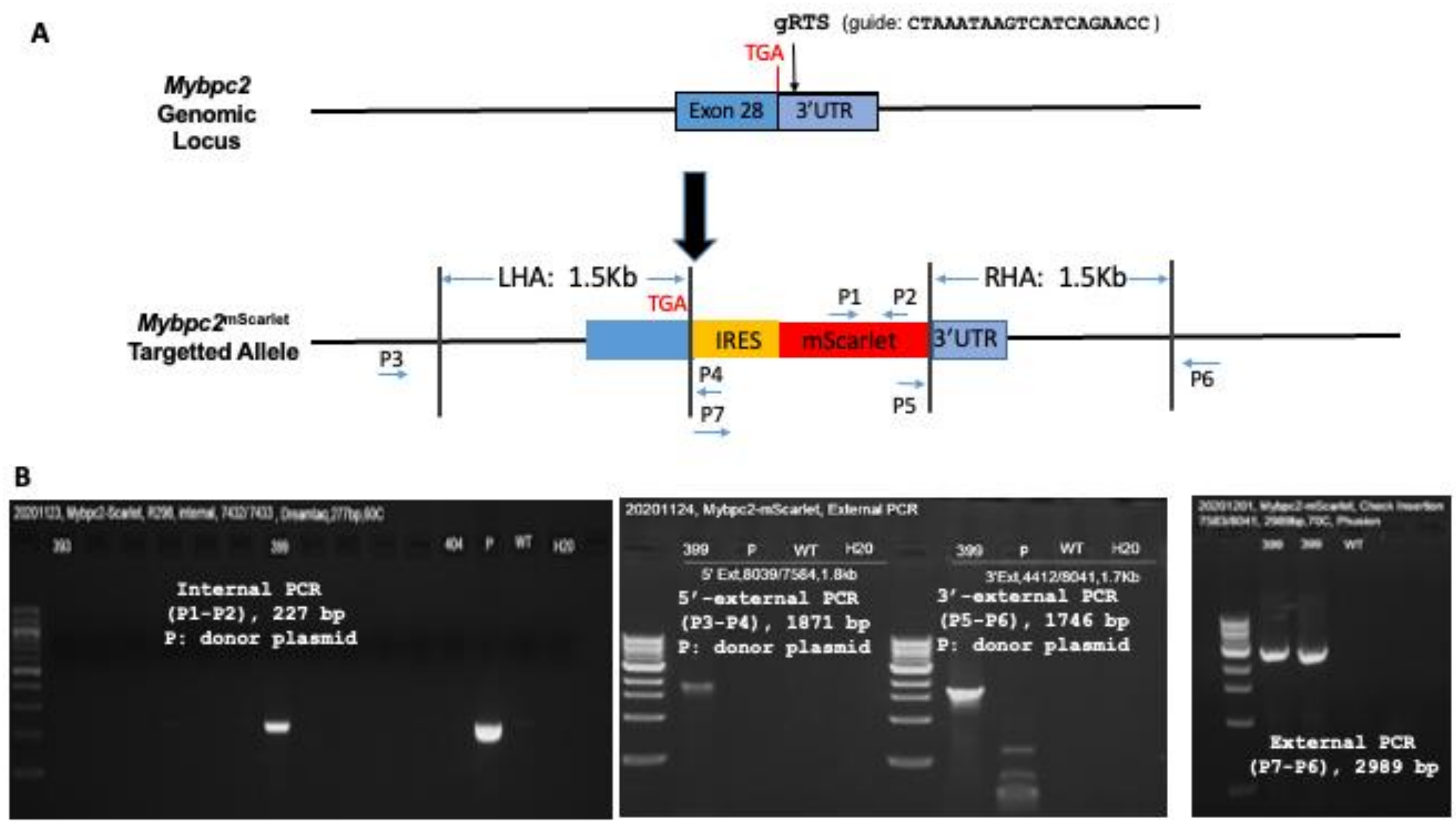

Supplementary Figure 2. Generation of *Mybpc2*$^{\mathbf{mScarlet}}$ mouse model. A. *Mybpc2*^{mScarlet} targeting strategy. B. Screening of founder mice for *Mybpc2*^{mScarlet} targeting.

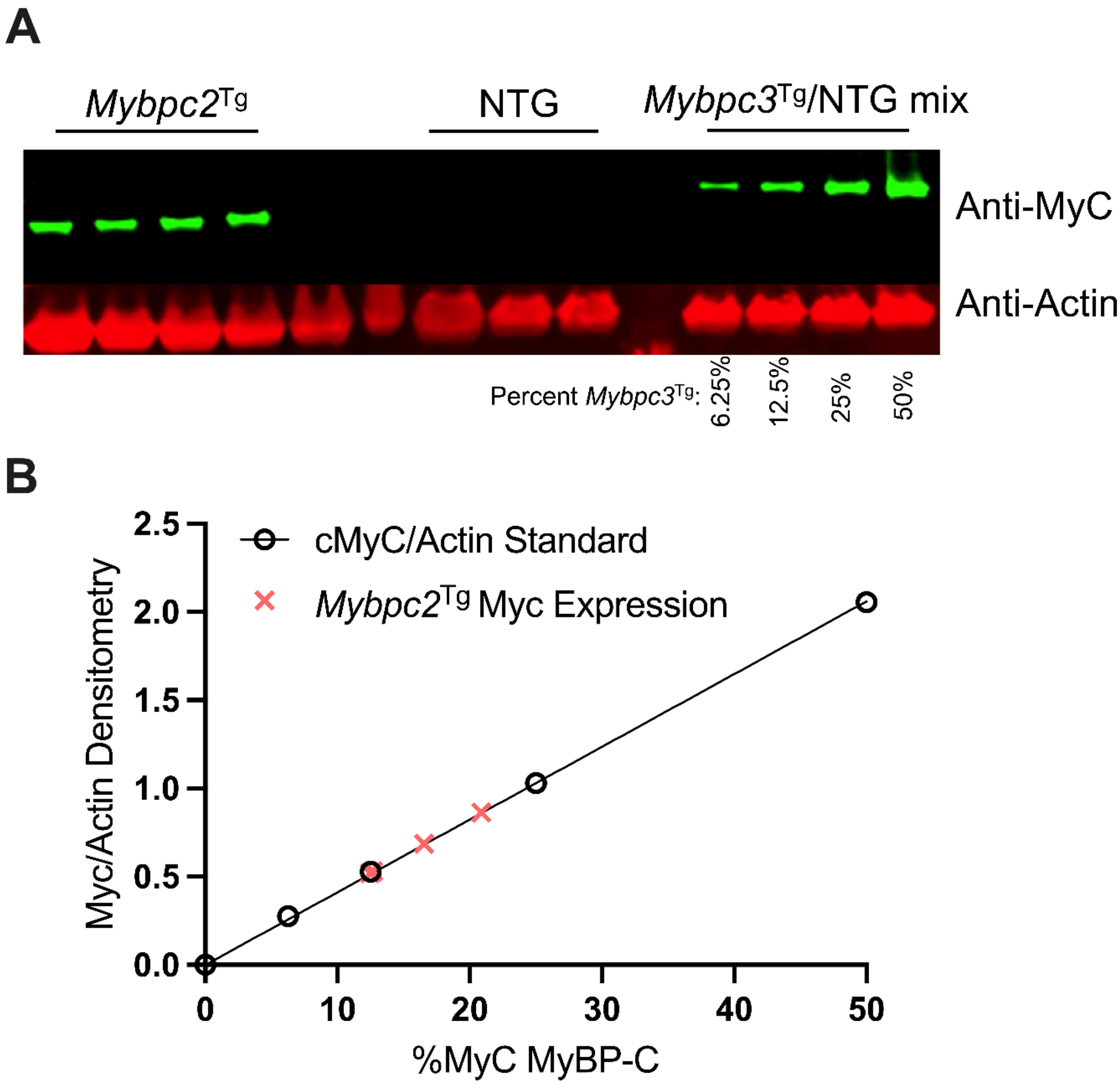

Supplementary Figure 3. Assessment of relative fMyBP-C amount. A. Western blot of *Mybpc2*^{Tg} myofilaments compared to a mixture of Myc tagged *Mybpc3*^{Tg} and NTG to assess total fMyBP-C levels. B. Myc-tagged fMyBP-C expression calculated from standard curve of Myc tagged *Mybpc3*^{Tg}.

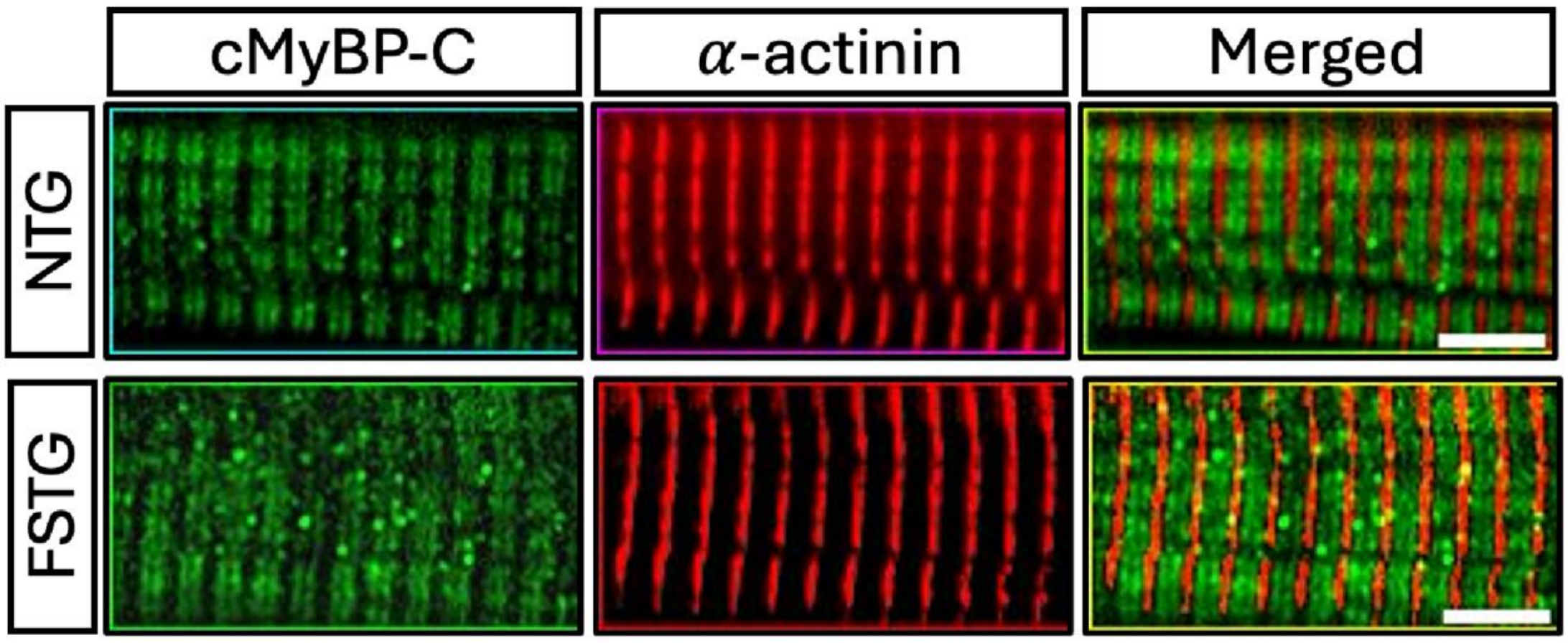

Supplementary Figure 4. Localization of cMyBP-C in NTG and FSTG mice. Isolated cardiomyocytes from NTG (non-transgenic) and FSTG (*Mybpc2*^{Tg}) mice immunostained with antibodies against cMyBP-C (green) and α-actinin (red), confirming proper localization of cMyBP-C between Z-disks, similar to fMyBP-C in FSTG mice, in both NTG and FSTG cardiomyocytes. Scale bar=5μm.

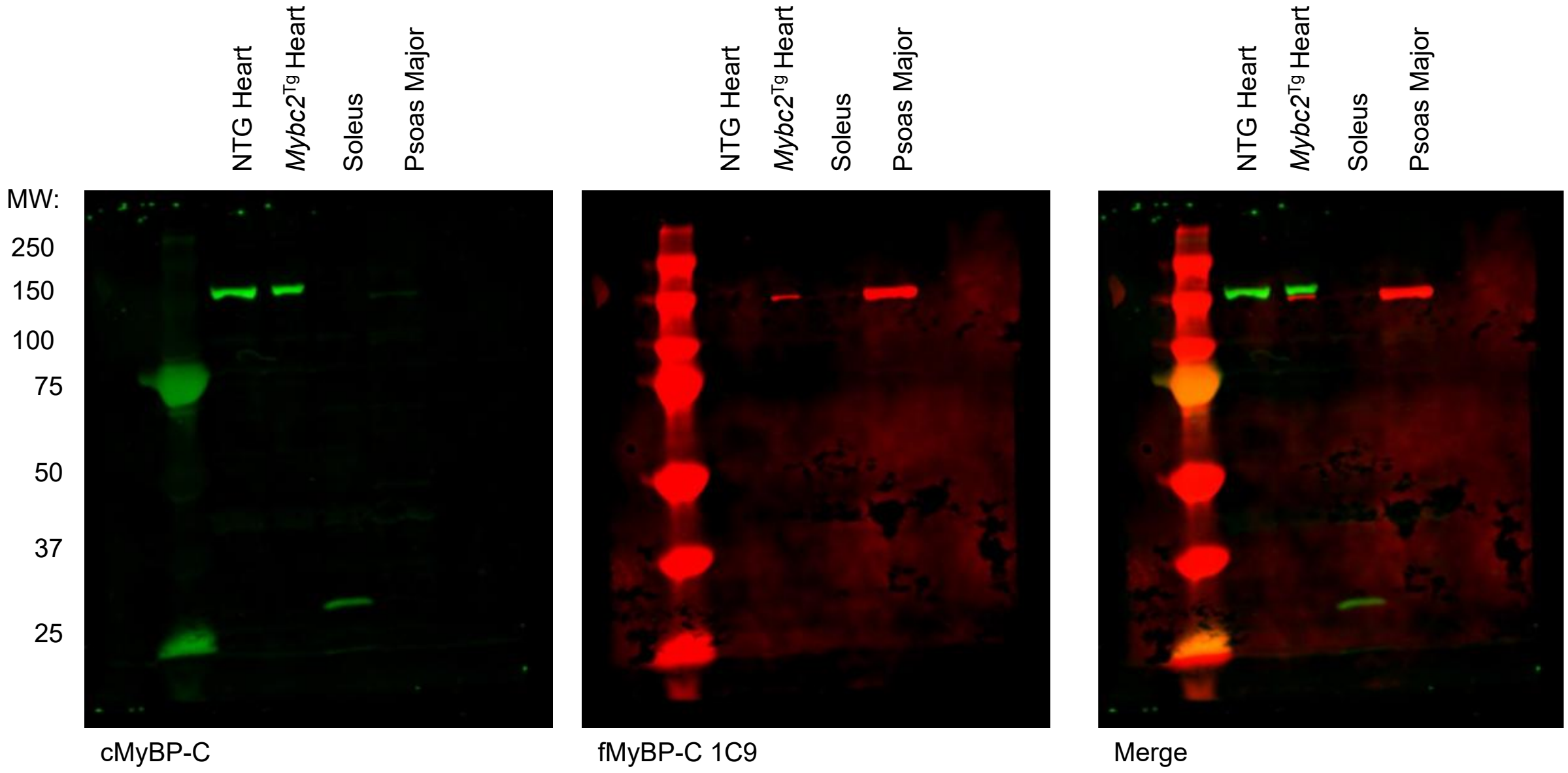

Supplementary Figure 5. Validation of custom 1C9 monoclonal fMyBP-C antibody.

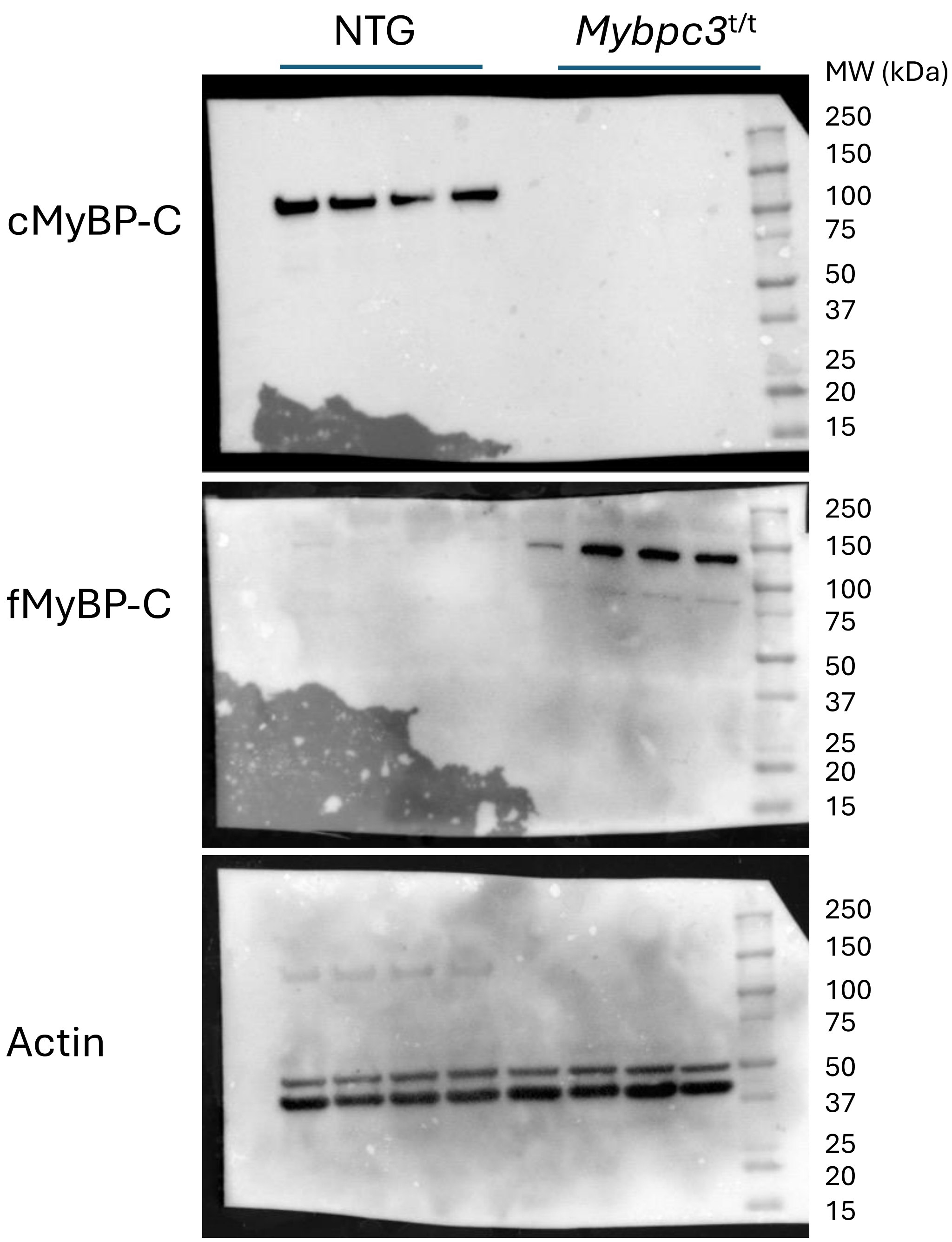

Supplementary Figure 6. Original Western Blots Corresponding to Figure 1B

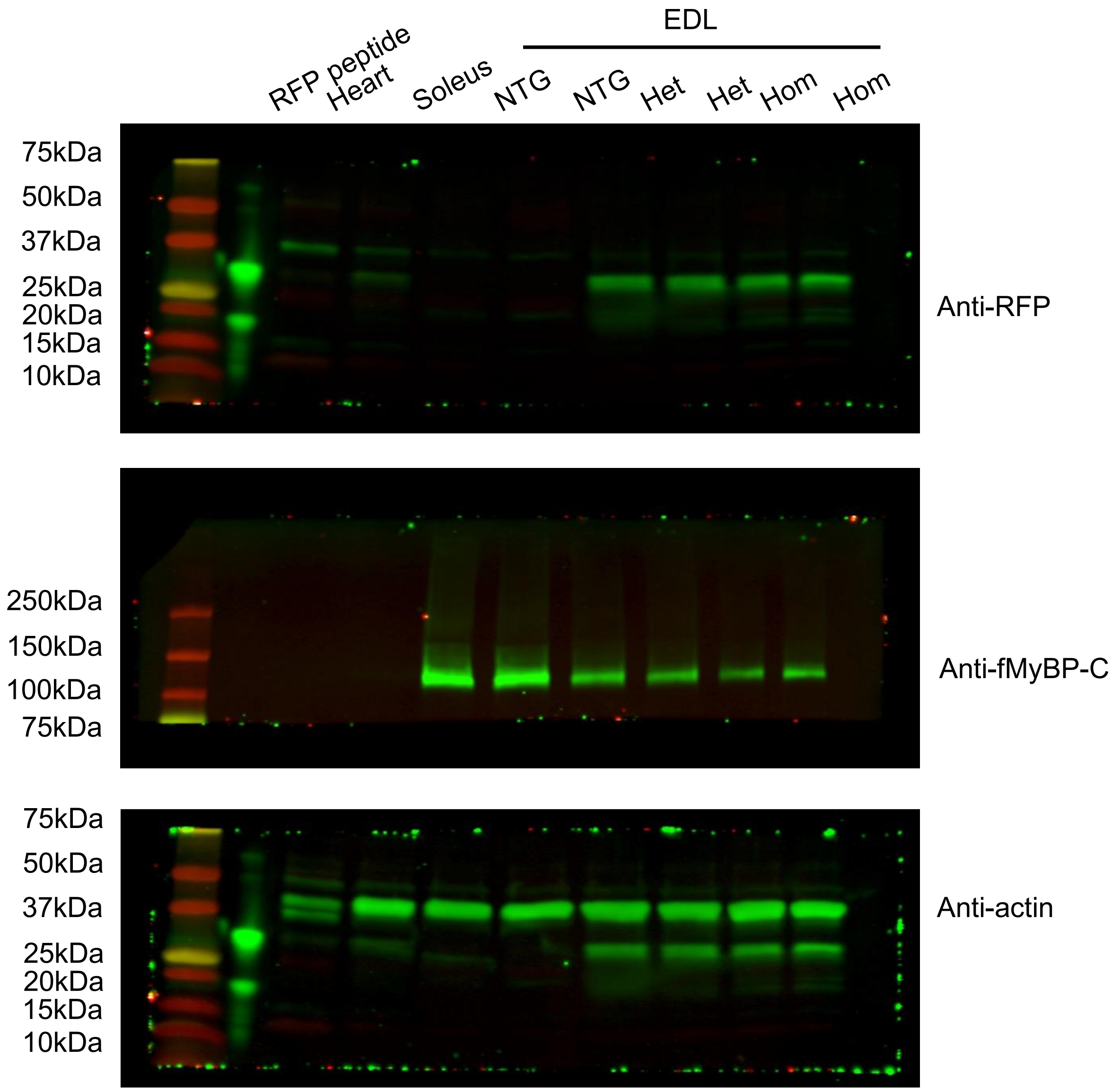

Supplementary Figure 7. Original Western Blots Corresponding to Figure 1H

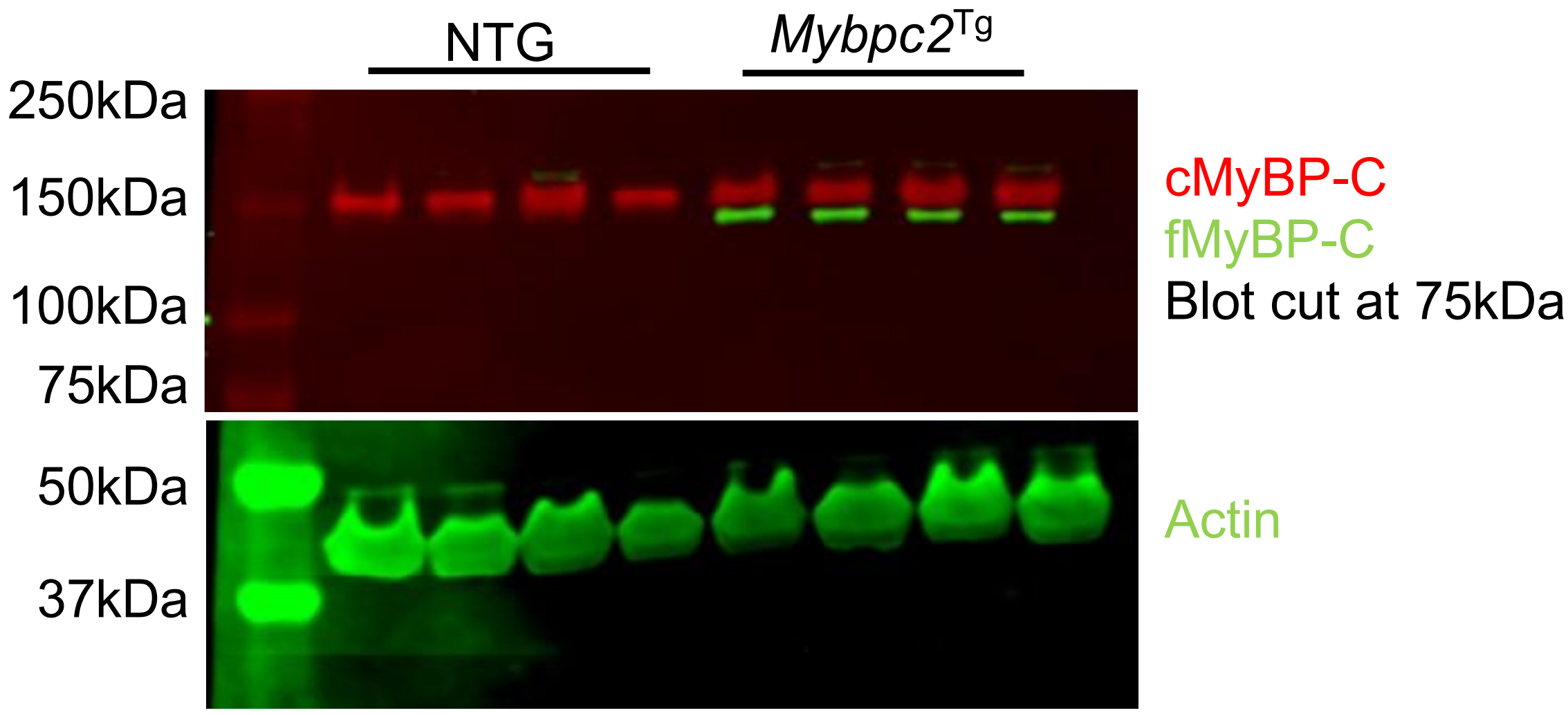

Supplementary Figure 8. Original Western Blots Corresponding to Figure 2B

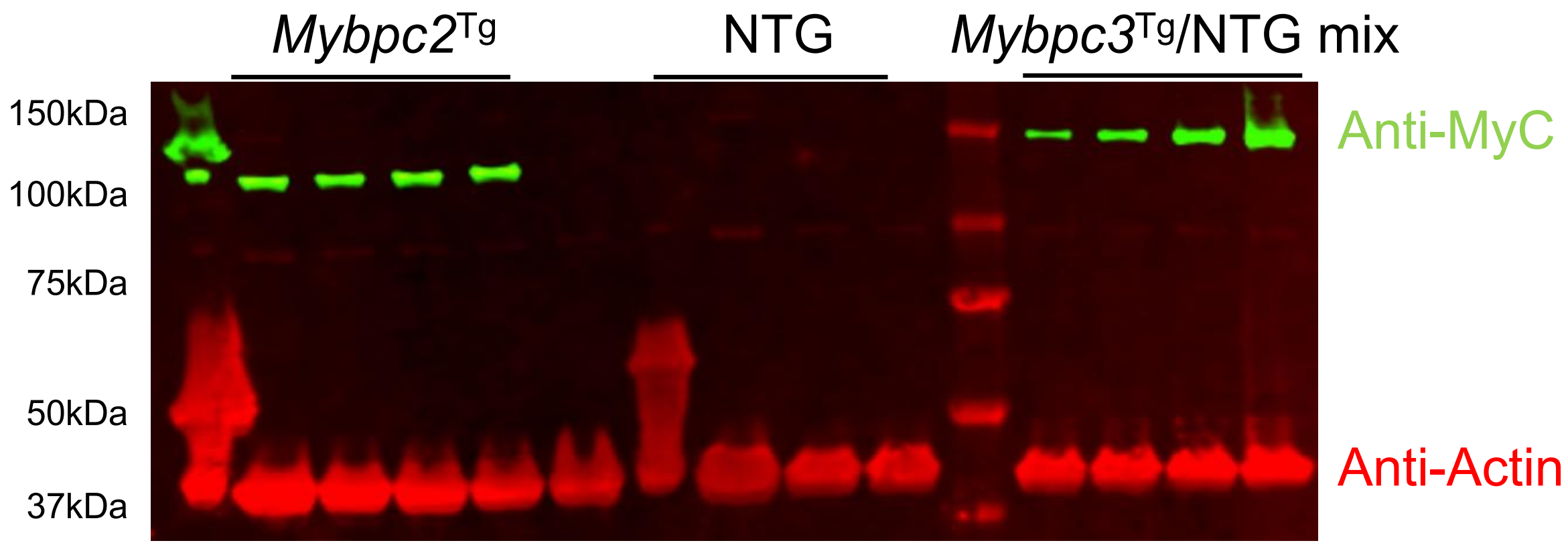

Supplementary Figure 9. Original Western Blots Corresponding to Figure 2D

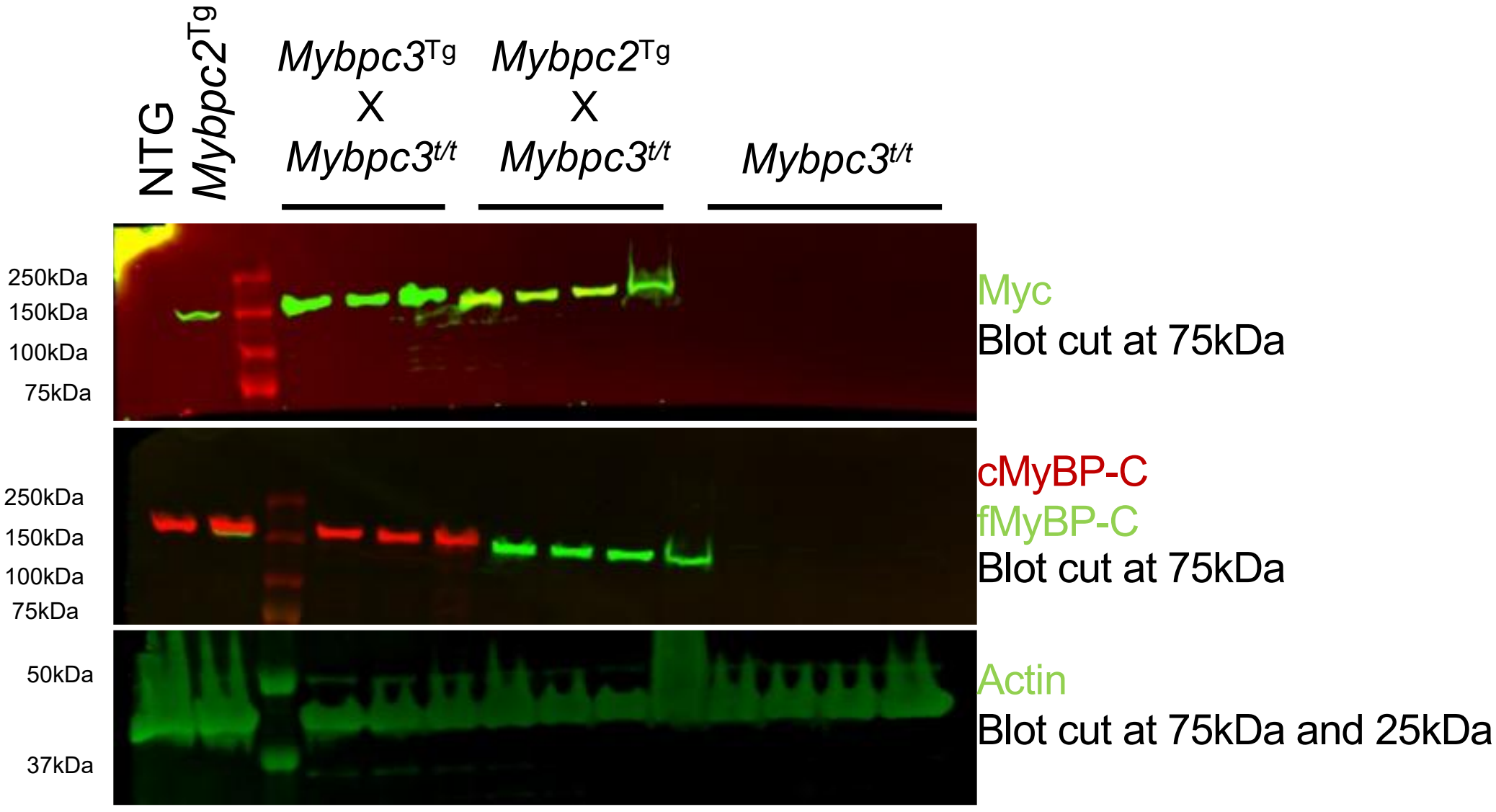

Supplementary Figure 10. Original Western Blots Corresponding to Figure 4A

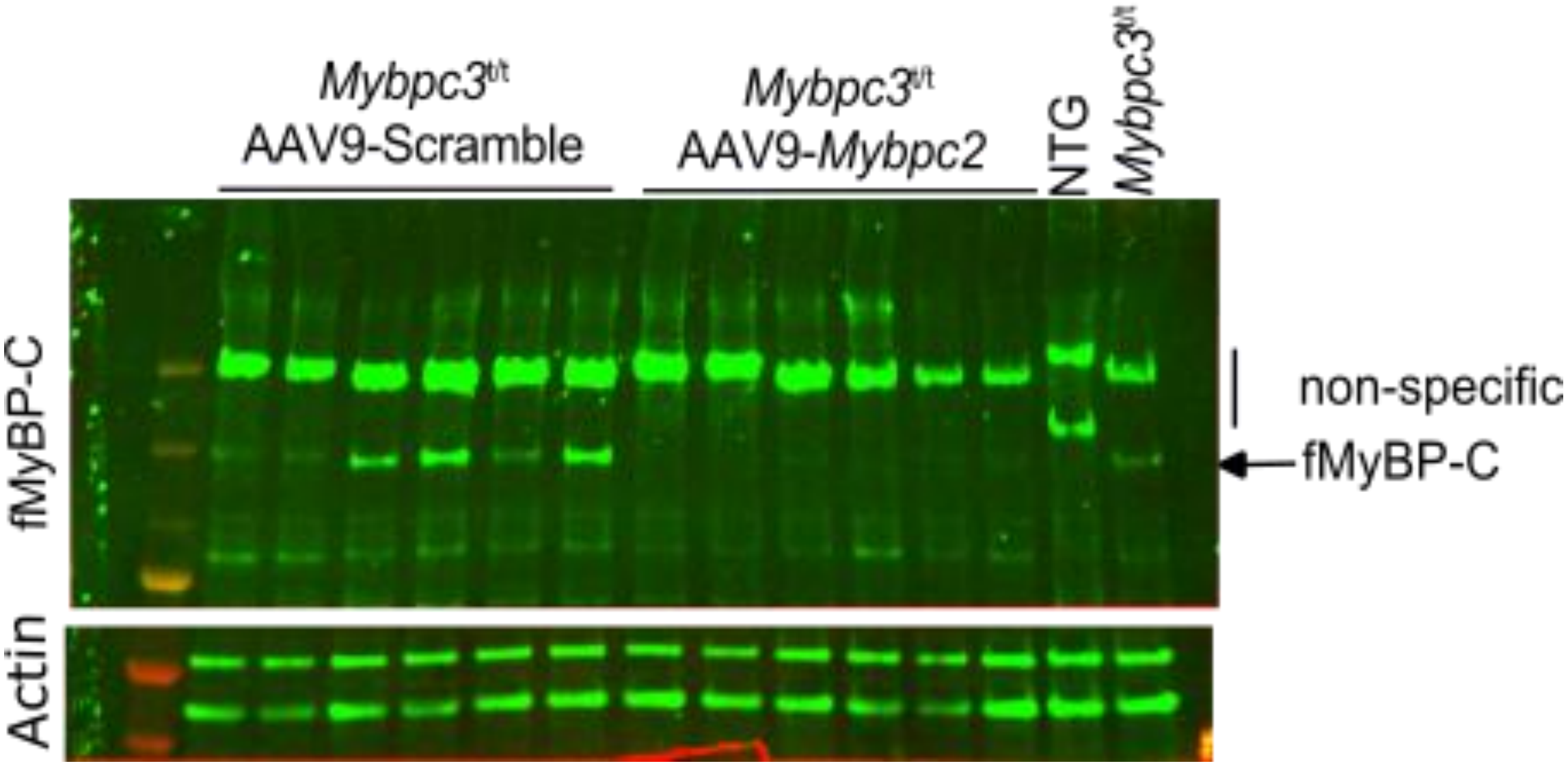

Supplementary Figure 11. Original Western Blots Corresponding to Figure 6C.

Supplementary Table 1. Echocardiographic parameters of *Mybpc2*^{Tg} and non-transgenic littermates

	NTG (n=5)		*Mybpc2*^{Tg} (n=6)		p-value
	Mean	S.E.M	Mean	S.E.M	
HR	469.40	17.90	459.50	13.49	0.6631
LVID;s (mm)	2.71	0.09	2.97	0.09	0.0653
LVID;d (mm)	3.93	0.06	4.29	0.06	0.0026
LV Vol;s (mm)	27.48	2.31	34.50	2.44	0.0697
LV Vol;d (mm)	67.31	2.45	83.23	2.68	0.0020
SV (μl)	39.85	1.51	48.70	1.80	0.0052
EF (%)	59.36	2.31	58.65	2.13	0.8267
FS (%)	31.16	1.55	30.91	1.52	0.9113
CO (ml/min)	18.79	1.16	22.32	0.77	0.0280
IVS;d (mm)	0.86	0.04	0.87	0.04	0.8596
IVS;s (mm)	1.23	0.05	1.14	0.08	0.4219
LVPW;d (mm)	0.98	0.06	0.96	0.04	0.7817
LVPW;s (mm)	1.20	0.06	1.23	0.06	0.7115

HR, Heart Rate; LVID, left ventricular internal diameter; LV Vol, left ventricular volume; SV, stroke volume; EF, ejection fraction; FS, fractional shortening; CO, cardiac output; IVS, interventricular septal thickness; LVPW, left ventricular posterior wall thickness; s, systolic measurement; d, diastolic measurement. p-values were calculated by unpaired students t-test.

Supplementary Table 2. Echocardiographic parameters of *Mybpc2*^{Tg}x*Mybpc3*$^{t/t}$ and *Mybpc3*$^{t/t}$ littermates

	Mybpc2^{Tg}x*Mybpc3*$^{t/t}$ (n=8)		*Mybpc3*$^{t/t}$ (n=9)		p-value
	Mean	S.E.M	Mean	S.E.M	
HR	399.84	17.85	446.44	20.56	0.086
LVID;s (mm)	4.56	0.17	4.02	0.10	0.021
LVID;d (mm)	5.26	0.15	4.80	0.09	0.030
LV Vol;s (mm)	96.60	8.92	71.88	4.93	0.026
LV Vol;d (mm)	134.02	9.22	108.37	5.16	0.035
SV (µl)	37.41	1.92	36.48	2.20	0.769
EF (%)	28.52	1.97	34.09	1.85	0.045
FS (%)	13.46	1.01	16.21	0.97	0.057
CO (ml/min)	15.07	1.22	16.22	1.41	0.481
IVS;d (mm)	0.91	0.06	1.12	0.07	0.011
IVS;s (mm)	1.01	0.07	1.29	0.08	0.005
LVPW;d (mm)	0.81	0.10	1.12	0.10	0.020
LVPW;s (mm)	0.91	0.11	1.29	0.13	0.011
Corr LVmass (mg)	157.55	10.89	199.17	12.49	0.032

HR, Heart Rate; LVID, left ventricular internal diameter; LV Vol, left ventricular volume; SV, stroke volume; EF, ejection fraction; FS, fractional shortening; CO, cardiac output; IVS, interventricular septal thickness; LVPW, left ventricular posterior wall thickness; Corr LVmass, corrected left ventricular mass; s, systolic measurement; d, diastolic measurement. p-values were calculated by unpaired students t-test.
