# Supplementary material for: Fast skeletal myosin binding protein-C expression exacerbates dysfunction in heart failure
Source: Basic Res Cardiol. 2026 Aug 5;121(5):1047–61. doi: 10.1007/s00395-026-01202-8 (PMC13593655; doi:10.1007/s00395-026-01202-8)
Supplement: Supplementary file 2 [file 395_2026_1202_MOESM2_ESM.pdf]

Appendix B: Materials and Methods

Fast skeletal myosin binding protein-C expression exacerbates dysfunction in heart failure

James W. McNamara[1,2,3*], Taejeong Song[1,4], Perwez Alam[5,#], Akhil Baby[1,4,6], Aleksandra Binek[7], Kalyani Ananthamohan[1,4], Rohit R. Singh[1§], Michelle L. Nieman[8], Marloes van den Berg[4], Brent M. Cernyar[4], Sheryl E. Koch[1], Malina J. Ivey[5], Thomas L. Lynch IV[9], James T. Pearson[10], Jack Rubinstein[1], J-P Jin[11€], John N. Lorenz[8], Jennifer E. Van Eyk[7,12], Onur Kanisicak[5†] and Sakthivel Sadayappan[1,4*]

[1]Center for Cardiovascular Research, Division of Cardiovascular Health and Disease, Department of Internal Medicine, University of Cincinnati, Cincinnati, OH, USA

[2]Murdoch Children's Research Institute, The Royal Children's Hospital, Parkville, Victoria 3052; Novo Nordisk Foundation Centre for Stem Cell Medicine (reNEW), Murdoch Children's Research Institute, Melbourne, Victoria, Australia

[3]Victor Chang Cardiac Research Institute, Victor Chang Cardiac Research Institute, Darlinghurst, New South Wales, Australia

[4]Department of Cellular and Molecular Medicine, Sarver Heart Center, University of Arizona College of Medicine, Tucson, Arizona, USA

[5]Department of Pathology and Laboratory Medicine, University of Cincinnati College of Medicine, Cincinnati, OH, USA.

[6]Department of Genetic Engineering, School of Biotechnology, Madurai Kamaraj University, Madurai, India

[7]Advanced Clinical Biosystems Research Institute, Smidt Heart Institute, Cedars-Sinai Medical Center, Los Angeles, California 90048, USA.

[8]Department of Pharmacology and Systems Physiology, University of Cincinnati College of Medicine, Cincinnati, OH, USA

[9]Department of Cell and Molecular Physiology, Loyola University Chicago, Maywood, IL, USA

[10]Department of Cardiac Physiology, National Cerebral and Cardiovascular Center Research Institute, Suita-shi, Osaka, Japan.

[11]Department of Physiology, Wayne State University School of Medicine, Detroit, MI, USA.

[12]Department of Biomedical Sciences, Cedars-Sinai Medical Center, Los Angeles, California 90048, USA.

[§]Current Address: Amgen, Discovery Protein Science, South San Francisco, CA 94080.

[#]Current Address: Department of Biomedical Sciences, University of Missouri, Columbia, Missouri-65211, USA.

[€]Current Address: Department of Phyiology and Biophysics, University of Illinois at Chicago, 835 S Wolcott Ave., Chicago, IL 60612.

* To whom correspondence should be addressed: j.mcnamara@victorchang.edu.au, sadayappan@arizona.edu

Animal Models

All experiments were conducted under institutional guidelines and approved by the University of Cincinnati (AM17-22-11-21-01) and the University of Arizona (2024-1236) Institutional Animal Care and Use Committee for Dr. Sadayappan and conform to the policies described in the Guide for the Use and Care of Laboratory Animals published by the National Institutes of Health. Mice were maintained on a 12-hour light/dark cycle at ambient room temperature and provided access to water and standard chow *ad libitum*. To generate cardiac specific over expression of *Mybpc2*, the *Mybpc2* transcript (NM_146189.3) was modified to include an in-frame Myc tag replacing the initiating methionine and flanking SalI restriction endonuclease restriction sites at each end. The resulting PCR product was cloned into the pBS-Sk2 + αMHC promoter vector (a gift from Dr. Jeffrey Robbins). After NotI backbone liberation of the vector backbone, the αMHC-*Mybpc2* transgene was injected into FvB/NJ zygotes. Founders and their offspring were screened with the following primers intron spanning primers: 5'-CTGTCAAGGATCTCCCAACTGGA-3' and 5'-GATATAAGTCTGACGAAGATGGCGGG-3'. *Mybpc3*$^{t/t}$ mice have been previously described [2, 8]. *Mybpc2*^{KO} mice were generated using CRISPR/Cas9 to induce a deletion between exons 6 and 7 of *Mybpc2*. Two synthetic gRNAs were used: 5'- GAAGCTCTCGAGGCTCTGAC-3' and 5'-GCAGGCGAGCTGGATTTCAG-3'. Founders were screened with primers 5'-GGAGGAAAGTGAATTGTGAATGC -3' and 5'-GAGGTGTTGGCACCTTGGCTAC-3'. A founder was identified with a 418bp deletion, corresponding to the production of a 157 amino acid protein product. *Mybpc2*^{mScarlet} mice were also generated using CRISPR/Cas9 to introduce an IRES-mScarlet sequence into the 3' UTR of *Mybpc2* using guide RNA sequence 5'-CTAAATAAGTCATCAGAACC-3'.

Transthoracic Aortic Constriction

Pressure overload of the LV was induced by TAC surgery, as previously described [3]. Briefly, animals were anesthetized using isoflurane, intubated, and ventilated. Following sternotomy, a 7-0 silk suture was used to tie down the aorta (between the innominate and left carotid arteries) around a 27-gauge needle (which was subsequently removed) to produce a constriction of uniform and reproducible diameter. Sham-operated animals were subjected to the identical surgical procedures, except that the suture was not tied to produce an occlusion.

RNA Isolation

RNA was isolated as previously described [2, 7]. Briefly, a small piece of tissue was homogenized in PureZOL reagent (BioRad). After incubation at room temperature for 5 minutes, phase separation was performed using chloroform. After centrifugation, the aqueous layer was precipitated with ice cold 70% (vol/vol) ethanol. Purification was then performed with using the Qiagen RNeasy mini kit (74104) following manufacturers procedures. RNA concentration and quality were measured using a Nanodrop and electrophoresis using a 3% agarose gel treated with 2% bleach.

Quantitative Polymerase Chain Reaction

cDNA was synthesized from 500ng RNA using the iScript cDNA synthesis kit (BioRad, 1708890). Following synthesis, cDNA was diluted 1:10 in nuclease free water and frozen at -20°C. Quantitative PCR was performed using) using the following primers.

Mybpc2:5'-CAGTGTGAGGCTGAACTCATT-3' 5'-GTCACCTTCTCATCAGACACTTC-3'
Mybpc3: 5'-GACACCTTCATCTTCCTTCTCTG-3' 5'-TGAGGAGTGGGTGTTTGATAAG-3'
Nppa: 5'-CTGGGACCCCTCCGATAGAT-3' 5'-TTCGGTACCGGAAGCTGTT-3'
Nppb: 5'-AAGGACCAAGGCCTCACAAA-3' 5'-ACAACAACTTCAGTGCGTTACA-3'
Actb: 5'-GGCTGTATTCCCCTCCATCG-3' 5'-CCAGTTGGTAACAATGCCATGT-3'
Myh7: 5'-GGAGAATCAGTCCATCCTCATC-3' 5'-CCCAATGGCGGCAATAAC-3'

Protein Isolation

Myofilament from left ventricular tissue were enriched as described [7]. Briefly, left ventricular tissue was minced and homogenized in F60 buffer (60mM KCl, 30mM imidazole, 2mM $MgCl_2$) containing protease inhibitor. After centrifugation the myofilament-containing pellet was treated with F60 buffer + 1% (vol/vol) Triton X-100 followed by centrifugation three times. Pellets were washed one time with F60 buffer before solubilization in protein solubilization buffer (BioRad 1632145). For total left ventricular homogenates, tissue was homogenized directly in protein solubilization buffer. Protein concentrations were measured using Bradford dye (Thermo Scientific 23200) using bovine serum albumin as a standard.

Western Blotting

Proteins were separated by SDS-PAGE using 4-15% Criterion tris-HCl acrylamide gels (BioRad). After electrophoresis, proteins were transferred onto nitrocellulose membranes by wet tank transfer at 300mA for 3 hours with ice cooling. Blots were blocked with 5% skim milk in TBS-T and incubated overnight in primary antibodies at 4°C with rocking. The following primary antibodies used were as follows: cMyBP-C (Rabbit polyclonal custom antibody against amino acids 2-14), fMyBP-C (custom mouse monoclonal raised against C1C2 region of mouse fMyBP-C, and Sigma Aldrich SAB2108180), RFP (rabbit polyclonal Rockland, 600-401-379), cMyc (rabbit polyclonal sigma C3956). Following 3 washes with TBS-T, blots were incubated in either Licor IRDye 800CW and 680RD secondary antibodies (1:10,000) or traditional HRP-conjugated secondary antibody for imaging on a Licor Odyssey DLx or BioRad Chemidoc, respectively. Band intensity was quantified using either Licor Image Studio or BioRad ImageLab.

Cardiomyocyte Isolation

Cardiomyocytes were isolated from mouse left ventricles using the Langendorff procedure as described [6]. Mice were injected with 0.15ml heparin (1000U/ml) and deeply anesthetized with euthasol (200mg/kg). After excision, hearts were mounted by the aorta on the Langendorff system for retrograde perfusion with a perfusion buffer consisting of NaCl (120 mm), KCl (5.4 mm), $MgSO_4$ (1.4

mm), NaH_2PO_4 (12 mm), $NaHCO_3$ (20 mm), 2,3-butadiene monoxime (10 mm), taurine (5 mm), and glucose (5.6 mm) at 37 °C for 3 min. After this, the hearts were perfused for 8-15 minutes with the same buffer but with 0.25mg Liberase (Roche Applied Science) and 12.5 μM $CaCl_2$ until the heart became flaccid. The ventricles were removed and dissociated by trituration, filtered, and allowed to pellet by gravity. These cardiomyocytes were resuspended in perfusion buffer with 0.5% BSA followed by sequential restoration of extracellular calcium to a concentration of 25 μM, 100 μM, 200 μM, and finally 1 mM calcium solution. *Mybpc2*^{mScarlet} cardiomyocytes were immediately observed under the fluorescent microscope to capture the endogenous mScarlet signal. Additionally, the remaining CMs were fixed and used for the immunofluorescent analysis.

Immunofluorescence

Cardiomyocytes and hearts were fixed in 4% paraformaldehyde. Hearts were then cryoprotected with sucrose and embedded in OCT. 10μm sections were cut using a cryostat. For immunofluorescent staining, tissue was first permeabilized with PBS containing 0.1% triton X-100, followed by blocking in 5% BSA in PBS containing 0.1% tween-20. Primary antibodies were incubated overnight at 4°C followed by washing and secondary incubation at room temperature for one hour. The following primary antibodies were used: cMyc (rabbit polyclonal sigma C3956), fMyBP-C (rabbit polyclonal Sigma Aldrich, SAB2108180), α-Actinin (mouse monoclonal, Sigma A7811), RFP (rabbit polyclonal Rockland, 600-401-379), cardiac troponin-T (mouse monoclonal, Fisher Scientific MS295P1). DAPI was used for nuclei visualization at a concentration of 1ug/ml. Cell membranes were visualized by staining with Wheat Germ Agglutinin labelled with Alexa Fluor488 (ThermoFisher).

Mass Spectrometry of tissue proteomics

Ventricular tissues were snap frozen in liquid nitrogen and stored until analysis. To increase proteome coverage, cardiac proteins were fractionated into cytoplasmic-, myofilament- and insoluble-enriched protein extracts as outlined [5]. 100μg of each fraction, quantified by BCA assay was reduced with 1mM TCEP and washed using S-trap mini spin columns (ProtiFi, Long Island, NY). Each fraction was then subjected to in-column trypsin digestion and eluted. Tryptic peptide concentration was determined using the Pierce Quantitative Colorimetric Peptide Assay Kit (Thermo Fisher Scientific). Samples were run in data independent acquisition mode (DIA) on the Orbitrap Fusion Lumos mass spectrometer equipped with an Easy Spray ion source and connected to Ultimate3000nanoLC system (Thermo Scientific) as described [1]. Raw proteomic data files were converted to mzml format using MSConvert. Files were searched using DIANN with label-free quantitation and match-between-runs approach against the complete mouse protein Uniprot database. Identified proteins based on peptides comprised of amino acid sequence unique to a protein, isoform identification was based on an isoform-specific peptide. The mass error tolerances were set at 15 ppm for both fragment and intact masses. The library-free identifications are set at <1% false discovery rate (FDR).

The mass spectrometry proteomics data have been deposited to the ProteomeXchange Consortium via the PRIDE [1] partner repository with the dataset identifier PXD047728.

Cardiac Function Measurement

Cardiac function was assessed non-invasively by echocardiography using the Vevo 2100 and the 30mHz MS-400 (FUJIFILM VisualSonics Inc., Toronto, Canada) probe as described [2]. Briefly, mice were anesthetized with 1-2% isoflurane and chest hair removed. Parasternal long- and short-axis B-Mode and M-Mode images were collected. Images were analyzed using Vevo Lab (FUJIFILM VisualSonics Inc., Toronto, Canada).

Cardiac function was assessed by invasive hemodynamics as previously described [10, 11]. Mice were anesthetized with ketamine-thiobutabarbital, and a tracheotomy was performed using polyethylene-90 tubing. The right femoral artery and vein were cannulated with polyethylene tubing to measure systemic arterial pressure and deliver drugs. To assess myocardial performance, closed chest animals were studied with a high fidelity 1.2F Scisense micromanometer tipped catheter placed retrograde across the aortic valve into the LV via the right carotid artery for pressure. Cardiovascular response to increasing doses of dobutamine were determined during 3-minute constant infusions (0.1 µl/min/gm body weight), and average values determined during the final 30 seconds of each infusion. Online heart rate, telemetry, LV developed pressure, +dP/dt, and -dP/dt were measured using PowerLab (ADInstruments).

Skinned Fiber Mechanics

The mechanical properties of chemically skinned fibers were assessed using the 1400A permeabilized fiber system (Aurora Scientific, Aurora, ON, Canada) using protocols identical to those described in [9, 12, 13]. Frozen left ventricular muscle was thawed and homogenized in a pCa 9.0 relaxing buffer (in mM: 7 EGTA, 100 BES, 0.017 CaCl2, 5.491 MgCl2, 5 DTT, 15 creatine phosphate, and 4.655 ATP; pH adjusted to 7.0 with KOH and ionic strength set to 180 with K-propionate. Triton X-100 was added to a concentration of 1% (vol/vol) to allow skinning overnight on ice. For AAV9 experiments, freshly isolated papillary muscles were used and skinned in an identical manner After washing with fresh relaxing buffer, muscle fibers were attached at each end with aluminum t-clips and mounted between the force transducer and length controller of the 1400A system. All measurements were taken at a sarcomere length of 2.1~2.3µm and 22°C.

Skinned Fiber mantATP Turnover Assays

Skinned fiber mantATP turnover assays were performed as described [9]. Left ventricle was skinned on ice for six hours in a buffer consisting of (in mM) 100 NaCl, 8 MgCl2, 5 EGTA, 5 K2HPO4, 5 KH2PO4, 3 NaN3, 5 ATP, 1 DTT, 20 BDM and 0.1% (v/v) Triton X-100 at pH 7, followed by overnight glycerination in a buffer consisting of (in mM): 120 K acetate, 5 Mg acetate, 5 EGTA, 2.5 K2HPO4, 2.5 KH2PO4, 50 MOPS, 5 ATP, 20 BDM, 2 DTT, and 50% (v/v) glycerol at pH 6.8, and after ~17 hours, new glycerinating solution was added. Tissue was stored at -20°C for use within 5 days. Small bundles were dissected and mounted in a flow cell and washed with rigor buffer consisting of (in mM) 120 K acetate, 5 Mg acetate, 5 EGTA, 2.5 K2HPO4, 2.5 KH2PO4, 50 MOPS, and 2 fresh DTT at pH

6.8. Next, rigor buffer containing 250µM mantATP (Sapphire Bioscience, Cat # NU-202L), a fluorescent analogue of ATP, was loaded into the cell chamber. After 60 seconds of imaging, the mant-ATP was replaced with a relaxing solution identical to the rigor solution but containing 4mM ATP. Imaging was carried out for up to 6 minutes. All SRX assays were performed at room temperature (approximately 22°C).

Development of anti-fMyBP-C monoclonal antibody

A short-term immunization procedure was applied to immunize an 8-week-old female Balb/c mouse. Recombinant C1C2 domain of fMyBP-C (a.a. 1-337) was expressed from cDNA cloned in pET28a+ plasmid with an N-terminal His-tag in transformed bacterial culture and purified using metal affinity chromatography. 50 mg of the purified C1C2 protein in 100 mL phosphate buffered saline (PBS) was mixed with an equal volume of Freund's complete adjuvant and injected intraperitoneally and intramuscularly. Ten days after the primary immunization, the mouse was intraperitoneally boosted daily with 100 mg of the C1C2 antigen in 200 mL PBS without adjuvant on two consecutive days. Two days after the final boost, spleen cells were harvested from the immunized mouse for fusion with Sp2mIL-6 mouse myeloma cells (ATCC CRL-2016) using 50% polyethaglycol$_{3500}$ (Invitrogen) containing 7.5% dimethyl sulfoxide as described previously. Hybridomas growing in 96 well culture plates were selected with HAT (0.1 mM hypoxanthine, 0.4 mM aminopterin, 16 mM thymidine) media containing 20% fetal bovine serum and screened using indirect enzyme-linked immunosorbent assay (ELISA) against plate-immobilized C1C2 protein using horseradish peroxidase (HRP)-labeled goat anti-mouse total immunoglobulin second antibody (Santa Cruz). Positive anti-fMyBP-C C1C2 antibody-secreting hybridoma clones were subcloned three times using the limiting dilution method [4] to establish stable cell lines. Culture supernatants of the hybridoma cells were collected and stored in aliquots after lyophilization.

Monoclonal antibody produced by one of the hybridoma lines (1C9) was used in the present study after validation of its specific recognition of fMyBP-C in Western blot of total protein extracts of skeletal and cardiac muscles (Fig. S5).

AAV-shRNA knockdown of Mybpc2

To assess the role of increased *Mybpc2* expression in *Mybpc3*$^{t/t}$ mice, an AAV9-*Mybpc2* shRNA was used to knockdown *Mybcp2* in the hearts of *Mybpc3*$^{t/t}$ mice. An AAV-shRNA vector was generated by VectorBuilder to target *Mybpc2* (sequence: TGGGTATACCTCCGGAGATTT), driven by a U6 promoter, with a distal mCherry reporter driven by a CAG promoter. A similar scramble shRNA plasmid (sequence: CCTAAGGTTAAGTCGCCCTCG) was also generated by VectorBuilder Inc (Chicago, IL), and both were packaged into AAV9 viral vector. At Postnatal day 4, *Mybpc3*$^{t/t}$ mice were injected via IP with 5e13vg/kg of either AAV9-*Mybp2* or scramble shRNA constructs diluted in PBS for a total injection volume of 50uL. To reduce the potential of maternal rejection, pups were gently rolled in soiled cage bedding to mask the scent of human-handling prior to being returned to the dam. For the following 3 hours post-injection, mice were closely monitored for recovery and assessed for any signs of adverse effects or distress according to University of Arizona Animal Care guidelines. Meeting

established health criteria, pups were weaned from the dam at 21 days of age and transferred to standard animal housing. Echocardiography was performed and analysed by a blinded observer at 8 weeks and 12 weeks of age, and mice were then culled. Cardiac tissue was collected for qPCR, Western blots, and skinned fiber mechanics. These experiments were not performed blinded.

Comparative expression analysis of *Mybpc2* and *Mybpc3* across multiple murine models of hypertrophic cardiomyopathy

Using publicly available RNA-Seq Data from NCBI GEO data sets, gene expression data were obtained from the Gene Expression Omnibus (GEO) database (*Mybpc3*$^{t/t}$: GSE335239 (this study), *Mybpc3*$^{\Delta 33-/-}$: GSE335235 (this study), *Mybpc3*$^{R946X+/-}$: GSE239872 [16], *TnT*$^{Tg\text{-}R92W}$: SRP083078 or PRJNA559482 [14, 15], and *Myh*$^{R403Q+/-}$: SRP083078 or PRJNA559482 [14, 15]. Normalized gene counts for *Mybpc2* and *Mybpc3* were extracted directly from the processed datasets and visualized to compare the expression levels of *Mybpc2* and *Mybpc3* across the selected models.

Statistical Analysis

All values are represented as mean ± SEM. Statistical analysis was performed using GraphPad Prism. Statistically significant differences between two groups were tested by an unpaired Student's t-test. One-way or two-way ANOVA was used when comparing more than two groups, followed by post hoc analysis with Tukey's multiple comparison test. All. Statistical significance was defined as $p < 0.05$.

References

1. Ai L, Binek A, Kreimer S, Ayres M, Stotland A, Van Eyk JE (2023) High-Field Asymmetric Waveform Ion Mobility Spectrometry: Practical Alternative for Cardiac Proteome Sample Processing. J Proteome Res 22:2124-2130 doi:10.1021/acs.jproteome.3c00027
2. Barefield DY, McNamara JW, Lynch TL, Kuster DWD, Govindan S, Haar L, Wang Y, Taylor EN, Lorenz JN, Nieman ML, Zhu G, Luther PK, Varró A, Dobrev D, Ai X, Janssen PML, Kass DA, Jones WK, Gilbert RJ, Sadayappan S (2019) Ablation of the calpain-targeted site in cardiac myosin binding protein-C is cardioprotective during ischemia-reperfusion injury. J Mol Cell Cardiol 129:236-246 doi:10.1016/j.yjmcc.2019.03.006
3. Green LC, Anthony SR, Slone S, Lanzillotta L, Nieman ML, Wu X, Robbins N, Jones SM, Roy S, Owens AP, 3rd, Aube J, Xu L, Lorenz JN, Blaxall BC, Rubinstein J, Benoit JB, Tranter M (2019) Human antigen R as a therapeutic target in pathological cardiac hypertrophy. JCI Insight 4 doi:10.1172/jci.insight.121541
4. Jin JP, Malik ML, Lin JJ (1990) Monoclonal antibodies against cardiac myosin heavy chain. Hybridoma 9:597-608 doi:10.1089/hyb.1990.9.597
5. Kane LA, Neverova I, Van Eyk JE (2007) Subfractionation of Heart Tissue. In: Vivanco F (ed) Cardiovascular Proteomics: Methods and Protocols. Humana Press, Totowa, NJ, p 87-90
6. Kumar M, Haghighi K, Kranias EG, Sadayappan S (2020) Phosphorylation of cardiac myosin-binding protein-C contributes to calcium homeostasis. J Biol Chem 295:11275-11291 doi:10.1074/jbc.RA120.013296
7. Lynch TLt, Kumar M, McNamara JW, Kuster DWD, Sivaguru M, Singh RR, Previs MJ, Lee KH, Kuffel G, Zilliox MJ, Lin BL, Ma W, Gibson AM, Blaxall BC, Nieman ML, Lorenz JN, Leichter DM, Leary OP, Janssen PML, de Tombe PP, Gilbert RJ, Craig R, Irving T, Warshaw DM, Sadayappan S (2021) Amino terminus of cardiac myosin binding protein-C regulates cardiac contractility. J Mol Cell Cardiol 156:33-44 doi:10.1016/j.yjmcc.2021.03.009
8. McConnell BK, Jones KA, Fatkin D, Arroyo LH, Lee RT, Aristizabal O, Turnbull DH, Georgakopoulos D, Kass D, Bond M, Niimura H, Schoen FJ, Conner D, Fischman DA, Seidman CE, Seidman JG (1999) Dilated cardiomyopathy in homozygous myosin-binding protein-C mutant mice. J Clin Invest 104:1235-1244 doi:10.1172/jci7377
9. McNamara JW, Singh RR, Sadayappan S (2019) Cardiac myosin binding protein-C phosphorylation regulates the super-relaxed state of myosin. Proc Natl Acad Sci U S A 116:11731-11736 doi:10.1073/pnas.1821660116
10. Rubinstein J, Lasko VM, Koch SE, Singh VP, Carreira V, Robbins N, Patel AR, Jiang M, Bidwell P, Kranias EG, Jones WK, Lorenz JN (2014) Novel role of transient receptor potential vanilloid 2 in the regulation of cardiac performance. Am J Physiol Heart Circ Physiol 306:H574-584 doi:10.1152/ajpheart.00854.2013
11. Sadayappan S, Gulick J, Osinska H, Martin LA, Hahn HS, Dorn GW, 2nd, Klevitsky R, Seidman CE, Seidman JG, Robbins J (2005) Cardiac myosin-binding protein-C phosphorylation and cardiac function. Circ Res 97:1156-1163 doi:10.1161/01.RES.0000190605.79013.4d

12. Singh RR, McNamara JW, Sadayappan S (2021) Mutations in myosin S2 alter cardiac myosin-binding protein-C interaction in hypertrophic cardiomyopathy in a phosphorylation-dependent manner. J Biol Chem 297:100836 doi:10.1016/j.jbc.2021.100836
13. Song T, McNamara JW, Ma W, Landim-Vieira M, Lee KH, Martin LA, Heiny JA, Lorenz JN, Craig R, Pinto JR, Irving T, Sadayappan S (2021) Fast skeletal myosin-binding protein-C regulates fast skeletal muscle contraction. Proc Natl Acad Sci U S A 118 doi:10.1073/pnas.2003596118
14. Vakrou S, Fukunaga R, Foster DB, Sorensen L, Liu Y, Guan Y, Woldemichael K, Pineda-Reyes R, Liu T, Tardiff JC, Leinwand LA, Tocchetti CG, Abraham TP, O'Rourke B, Aon MA, Abraham MR (2018) Allele-specific differences in transcriptome, miRNome, and mitochondrial function in two hypertrophic cardiomyopathy mouse models. JCI Insight 3 doi:10.1172/jci.insight.94493
15. Vakrou S, Liu Y, Zhu L, Greenland GV, Simsek B, Hebl VB, Guan Y, Woldemichael K, Talbot CC, Aon MA, Fukunaga R, Abraham MR (2021) Differences in molecular phenotype in mouse and human hypertrophic cardiomyopathy. Sci Rep 11:13163 doi:10.1038/s41598-021-89451-6
16. Wu S, Yang P, Geng Z, Li Y, Guo Z, Lou Y, Zhang S, Xiong J, Hu H, Guo X, Pu WT, Zhang Y, Zhu D, Zhang B (2024) Base editing effectively prevents early-onset severe cardiomyopathy in Mybpc3 mutant mice. Cell Res 34:327-330 doi:10.1038/s41422-024-00930-7
